# Germline copy number variations in *BRCA1/2* negative families: Role in the molecular etiology of hereditary breast cancer in Tunisia

**DOI:** 10.1371/journal.pone.0245362

**Published:** 2021-01-27

**Authors:** Maroua Boujemaa, Yosr Hamdi, Nesrine Mejri, Lilia Romdhane, Kais Ghedira, Hanen Bouaziz, Houda El Benna, Soumaya Labidi, Hamza Dallali, Olfa Jaidane, Sonia Ben Nasr, Abderrazek Haddaoui, Khaled Rahal, Sonia Abdelhak, Hamouda Boussen, Mohamed Samir Boubaker

**Affiliations:** 1 Laboratory of Biomedical Genomics and Oncogenetics, LR16IPT05, Institut Pasteur de Tunis, University of Tunis El Manar, Tunis, Tunisia; 2 Laboratory of Human and Experimental Pathology, Institut Pasteur de Tunis, Tunis, Tunisia; 3 Medical Oncology Department, Abderrahman Mami Hospital, Faculty of Medicine Tunis, University Tunis El Manar, Tunis, Tunisia; 4 Department of Biology, Faculty of Science of Bizerte, University of Carthage, Jarzouna, Tunisia; 5 Laboratory of Bioinformatics, Biomathematics and Biostatistics, LR16IPT09, Institut Pasteur de Tunis, University of Tunis El Manar, Tunis, Tunisia; 6 Surgical Oncology Department, Salah Azaiez Institute of Cancer, Tunis, Tunisia; 7 Department of Medical Oncology, Military Hospital of Tunis, Tunis, Tunisia; German Cancer Research Center (DKFZ), GERMANY

## Abstract

Hereditary breast cancer accounts for 5–10% of all breast cancer cases. So far, known genetic risk factors account for only 50% of the breast cancer genetic component and almost a quarter of hereditary cases are carriers of pathogenic mutations in *BRCA1/2* genes. Hence, the genetic basis for a significant fraction of familial cases remains unsolved. This missing heritability may be explained in part by Copy Number Variations (CNVs). We herein aimed to evaluate the contribution of CNVs to hereditary breast cancer in Tunisia. Whole exome sequencing was performed for 9 *BRCA* negative cases with a strong family history of breast cancer and 10 matched controls. CNVs were called using the ExomeDepth R-package and investigated by pathway analysis and web-based bioinformatic tools. Overall, 483 CNVs have been identified in breast cancer patients. Rare CNVs affecting cancer genes were detected, of special interest were those disrupting *APC2*, *POU5F1*, *DOCK8*, *KANSL1*, *TMTC3* and the mismatch repair gene *PMS2*. In addition, common CNVs known to be associated with breast cancer risk have also been identified including CNVs on *APOBECA/B*, *UGT2B17* and *GSTT1* genes. Whereas those disrupting *SULT1A1* and *UGT2B15* seem to correlate with good clinical response to tamoxifen. Our study revealed new insights regarding CNVs and breast cancer risk in the Tunisian population. These findings suggest that rare and common CNVs may contribute to disease susceptibility. Those affecting mismatch repair genes are of interest and require additional attention since it may help to select candidates for immunotherapy leading to better outcomes.

## Introduction

Breast cancer is the most common malignancy in women worldwide with approximately 2.09 million new cases diagnosed per year [1]. It is estimated that 5–10% of all breast cancers are hereditary cases [2, 3]. Family based linkage, gene re-sequencing as well as genome wide association studies allowed the identification of high, moderate and low penetrant variants that collectively explained only half of the breast cancer genetic component [3, 4]. Thus, the genetic background of a substantial part of hereditary cases are yet to be discovered. Copy number variations (CNVs), typically defined as a gain or a loss of DNA sequences larger than 50 bp compared to a reference genome [5], might contribute to the remaining genetic basis of breast cancer risk [6]. Several CNVs have already been identified as associated with many diseases including complex disorders such as cancer [7]. CNVs may contribute to disease development through their impact on gene expression and protein structure. Indeed, deleterious CNVs in cancer patients have been observed in more than 30% of highly penetrant cancer-predisposing genes, including *BRCA1*, *BRCA2*, *APC*, as well as mismatch repair (MMR) genes [7, 8].

Germline CNVs represent 4 to 28% of all inherited *BRCA* mutations depending on the study population [9]. Pathogenic CNVs are more frequent in *BRCA1* than *BRCA2* and reach respectively 27% and 8% of *BRCA* genetic variations. This may be explained by the higher number of *Alu* sequences in *BRCA1*, and also by the homologous recombination events that occur between *BRCA1* and its pseudogene [10, 11]. The highest contribution of *BRCA1* CNVs was reported in the Dutch population in which 27% to 36% of all germline *BRCA1* mutations are CNVs [6]. In the Tunisian population, the contribution of *BRCA* CNVs to breast cancer susceptibility is not well defined. A unique report was published describing exon 5 deletion and exon 20 duplication in *BRCA1* identified each in one patient [12]. Furthermore, several studies have been conducted in *BRCA* negative breast cancer patients and have led to the identification of rare candidate CNVs that might contribute to breast cancer susceptibility. [3, 8, 13–15]. Common CNVs are also expected to play a role in cancer etiology. It was shown that approximately 40% of cancer-related genes are disrupted by a common CNV. These common cancer CNVs, and by analogy with common cancer SNPs, are thought to confer, each, only a minor increase in the disease risk but when considered collectively they may lead to a substantially elevated risk [16].

The association between common germline CNVs and breast cancer risk was assessed only in a few reports. Recently, a genome wide association study in Chinese population revealed an association between a common copy number deletion in *APOBEC3* loci and breast cancer risk with 1.31 and 1.76-folds increased risk associated with one copy deletion and two copy deletion, respectively [17]. This finding was subsequently validated in Caucasian population [18]. Moreover, other common CNVs were found to be associated with breast cancer risk through whole genome CNV genotyping studies including those disrupting *OR4C11*, *OR4P4*, *OR4S2* and *UGT2B17* genes [4]. These results were replicated in the study of Kumaran et al,.2017 which revealed the association of 200 common CNVs (frequency >10%) with breast cancer risk of these, 21 CNVs were also associated with disease prognosis. Those disrupting *ZFP14*, *JAK1*, *LPA* and *PDGFRA* genes were found to be associated with both recurrence-free survival and overall survival [19]. The association between CNVs and disease prognosis in breast cancer patients has also been explored in earlier studies where CNVs in the drug metabolism genes *GSTT1* and *GSTM1 w*ere found to predict treatment outcome [20]. The association between other metabolizing enzymes such as CYP2D6 and SULT1A1 and the clinical response toward tamoxifen therapy in breast cancer patients have been also evaluated in several reports [21, 22].

So far, several techniques have been used to characterize CNVs such as multiplex ligation-dependent probe amplification (MLPA), real-time PCR and genomic arrays [7]. Nevertheless, thanks to advances made in sequencing technologies such as next generation sequencing (NGS), which generate millions of sequences of the same target genomic region, it is now possible to detect CNVs from NGS data using the appropriate bioinformatics tools. These latter usually applied a read depth approach based on counting the number of reads aligned to a particular region of the human genome [10, 23].

Here, we used whole exome data to evaluate the contribution of germline CNVs to breast cancer risk in Tunisian patients who were negative for pathogenic mutations in known breast cancer susceptibility genes.

## Materials and methods

### Patients

The studied cohort included 9 patients with a strong family history of breast cancer referred from the Departments of Medical Oncology of Abderrahman Mami Hospital, Surgical Oncology of Salah Azaiez Institute and Medical Oncology of the Military Hospital of Tunis. In addition, 10 non-affected unrelated individuals were included as matched controls for CNVs detection. Written informed consent was obtained from all study participants. The present study was conducted in accordance with the ethical standards of Helsinki declaration and approved by the biomedical ethics committee of Institut Pasteur de Tunis (2017/16/E/Hôpital A-M).

### DNA isolation

Genomic DNA was isolated from peripheral blood, collected on EDTA, by the salt precipitation method [24]. DNA quantity and purity were evaluated using a NanoDrop™ spectrophotometer.

### Whole Exome Sequencing (WES)

Whole Exome Sequencing was performed on breast cancer patients and control individuals. Samples were prepared according to Agilent's SureSelect Protocol Version 1.2 and enrichment was carried out according to Agilent SureSelect protocols. Paired-end (2 × 100) sequencing was performed on enriched samples on the Illumina HiSeq2000 platform using TruSeq v3 chemistry. Data were analyzed as described elsewhere [25]. In order to assess the quality of sequencing and to ensure that target regions are well covered, coverage analysis was performed using GATK [26] and VarAFT.2.10 [27] softwares.

### Copy number variations detection and analysis

CNVs were called from WES data using the ExomeDepth R package that uses read depth data to call CNVs from exome sequencing experiments. Each tested exome was compared to an optimized set of the control exomes that had been generated by identical laboratory and computational procedures. ExomeDepth presumes that the CNV of interest is absent from the aggregate reference set [28]. Analysis was performed using the hg19 assembly as a human reference genome. Identified CNVs were annotated using the AnnotSV program which is designed for annotating and ranking Structural Variations (SVs) [29]. This program provides several relevant annotations including the computed allelic frequency relative to overlapping CNVs from the Database of Genomic Variants (DGV), the 1000 genomes project and the Deciphering Developmental Disorders (DDD) study that contain a catalogue of SVs of control individuals from worldwide populations [30, 31]. It also reports frequencies of overlapping CNVs from gnomAD and I.M. Hall's lab [32]. In addition to these annotations, this tool also provides a systematic CNVs classification based on the same type of categories delineated by the American College of Medical Genetics and Genomics (ACMG) (Class 1 = benign; Class 2 = likely benign; Class 3 = VOUS (variant of unknown significance); Class 4 = likely pathogenic; Class 5 = pathogenic). In order to prioritize clinically relevant CNVs, we have first eliminated those considered as common. Indeed, a CNV was thought to be common if at least 70% of this CNV is overlapped with a documented CNV from the DGV, the 1000 genomes database, the DDD study data control sets, gnomAD or the I.M. Hall's lab and it has a frequency ≥ 1%. Otherwise, the called CNV is considered as rare. Subsequently, only CNVs classified as likely pathogenic or pathogenic were kept for further analysis.

In addition, we have searched published data on common CNVs and breast cancer risk to assess the possible contribution of this type of variations to hereditary breast cancer in the studied cohort.

### Gene set enrichment analysis and biological pathways investigation

Overrepresentation enrichment analysis was conducted using EnrichR, a bioinformatics web-based tool that contains a large collection of more than 100 gene set libraries [33]. Enriched pathways were visualized using ClueGO, a cytoscape plug-in that allows the visualization of the non-redundant biological terms for large clusters of genes in a functionally grouped network [34].

We investigated the biological and functional features of genes contained within CNVs classified as likely pathogenic and pathogenic using different online databases: 1) Network of Cancer Genes version 6.0 to identify genes associated with malignancy [35], 2) Web-based Gene Set Analysis Toolkit V2 (WebGestalt2) to reveal common functions of the gene products [36], 3) Kyoto Encyclopedia of Genes and Genomes (KEGG) Mapper–Search Disease tool for searching disease genes in the KEGG DISEASE database [37], 4) The Database for Annotation, Visualization and Integrated Discovery (DAVID) v6.8 which provides a comprehensive set of functional annotation tools for investigators to understand biological meaning behind large list of genes and to identify genomic loci associated with genetic disorders including cancer [38]. Moreover, and to select genes likely associated with malignancy a gene disease association (GDA) network was generated using the DisGeNET Cytoscape App. This latter interrogates the DisGeNET database, which integrates gene-disease associations from literature and from various expert curated databases [39].

## Results

In the current study, we performed whole exome sequencing for 9 *BRCA* negative breast cancer cases and 10 matched controls with the aim to assess the contribution of germline CNVs to hereditary breast cancer in the Tunisian population. The mean age at diagnosis for breast cancer patients included in this study was 43.9 years old (29–60 years) and family history of breast and/or ovarian cancer was present in all cases. Table 1 summarizes the epidemio-clinicopathological characteristics of these patients. WES data analysis showed no deleterious point mutations on all known breast cancer susceptibility genes. Coverage analysis demonstrated that target regions are well covered, eliminating the possibility of false negative results (Additional data are given in S1 Table). These findings led us to hypothesize that other forms of variations such as CNVs may account for disease susceptibility.

**Table 1:** Epidemiological and clinicopathological features of the investigated patients.

Patient ID	Age at diagnoses	Family History	Histology	Histological grade SBR	Ki67 index (%)	TNM	Lymph node status	Tumor size (mm)	ER	PR	HER2 overexpression	Treatment	Outcome
**BC1-1**	43	3 BC	IDC	II	NA	NA	NA	22	+	+	NA	Lumpectomy	CBC within 5 years, Grade III triple negative carcinoma
		2 Lung cancer										CT	
		1 cerebral cancer										RT	
		1 lymphoma										HT(TAM)	
**BC1-8**	56		IDC	I	NA	NA	NA	NA	+	+	NA	NA	In remission
**BC19**	49	3 BC	IDC	II	NA	T4bN3M0	24N-	60	-	-	NA	CT(6 FEC)	In remission until 2012
												Patey	
												RT	
**BC22**	29	1 BC	IDC	III	NA	T3N1M1	2N+/15N	47	+	+	NA	CT(4 FEC)	Hepatic relapse 16 months after the end of the CT
												Patey	
		1 Pancreatic cancer										RT	
		1 Skin cancer										HT (TAM)	
**BC37**	34	1 BC	IDC	III	30	T2N0M0	7N+/19N	40	+	+	No	Patey	In remission
												CT (3FEC/3DOC)	
		1 Lung cancer										RT	
												HT (TAM)	
**BC39**	60	1 BC	IDC	III	50	T2N0M0	11N-	20	+	+	No	Patey	In remission
		1 colon cancer											
		1 uterus cancer											
		1 larynx										CT (3FEC/3DOC)	
		1 testicular cancer										HT (Anastrozole)	
**BC40**	37	1 BC	IDC	I	22	T1N0M0	12N-	20	+	+	Yes	Lumpectomy	In remission
												CT (3FEC/3DOC)	
												RT	
		1 gastric cancer										Trastuzumab	
												HT (TAM)	
**BC47**	48	1 OC	IDC	II	<10	T2NxMx	26N+/29N	40	-	-	Yes	Patey	Pleural and hepatic relapse 16 months after the end of the CT.
		1 gastric cancer										CT (3FEC/3DOC)	Patient died at 50 years old
		1 lung cancer										RT	
**BC52**	42	3 BC	IDC	II	20	T2N0M0	12N-	17	+	+	No	TCA	In remission
		2 prostate cancer										CT (AC)	
		1 uterus cancer										RT	
		1 hepatic cancer										HT (TAM)	

**Abbreviations:** AC: Adriamycin and Cyclophosphamide; BC = breast cancer; CT = chemotherapy; ER = Estrogen Receptor; FEC = 5-Fluorouracil-Epirubicin-Cyclophosmamide; HER2 = Human Epidermal Growth Factor Receptor2; IDC = Invasive Ductal Carcinoma; NA = not available; OC = Ovarian cancer; PR = Progesterone Receptor; RT = Radiotherapy; TAM = Tamoxifen; DOC = Docetaxel;; SBR: Scarff, Bloom et Richardson. CBC: Contralateral Breast Cancer.

### CNVs distribution

**Fig 1:**
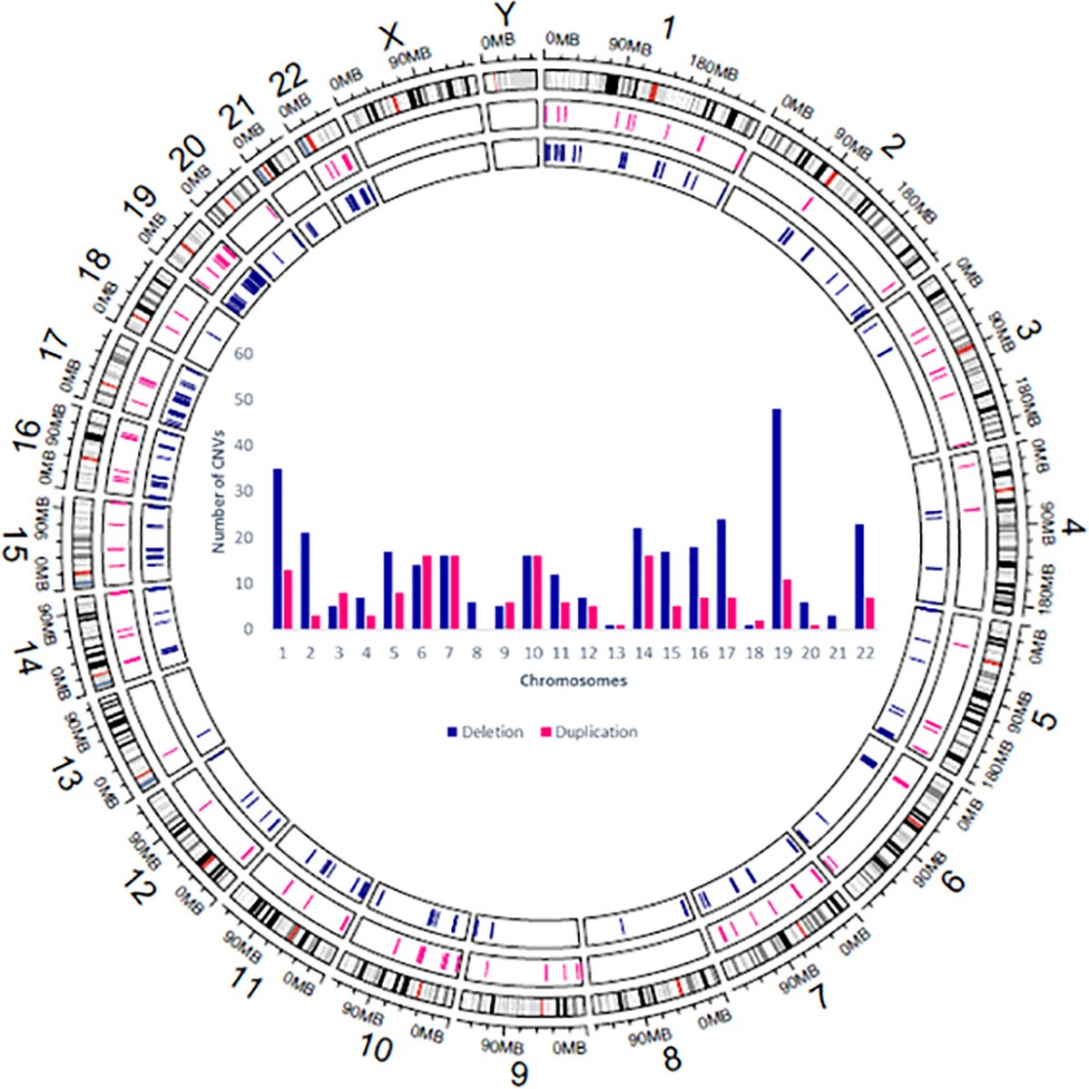
Chromosomal distribution of CNVs among the studied breast cancer patients. The Circos plot generated using Circlize R package [40] summarizes all CNV regions identified in the 9 breast cancer patients. On each chromosome the first track indicates localization of deletions and the second track designates localization of duplications. The central Histogram illustrates the number of deletions and duplications detected on each chromosome.

CNV analysis revealed a total of 483 Copy Number Variations affecting 524 coding genes and consisting of 324 deletions and 159 duplications with an average size of 30.6 kb ranging from 55bp to 734.284kp. The mean size of duplications was significantly greater than that of deletions 51.96kb vs 20.13kb (p-value: 0.0001 (Welch Two Sample t-test)). Moreover, CNVs were found to be unequally distributed among chromosomes and were more frequent in pericentromeric and subtelomeric regions. For deletions, chromosomes 1, 17, 19 and 22 showed the highest proportion of CNVs whereas for duplications the highest number of CNVs were found in chromosomes 6, 7, 10 and 14 (Fig 1).

### Gene set enrichment analysis

**Fig 2:**
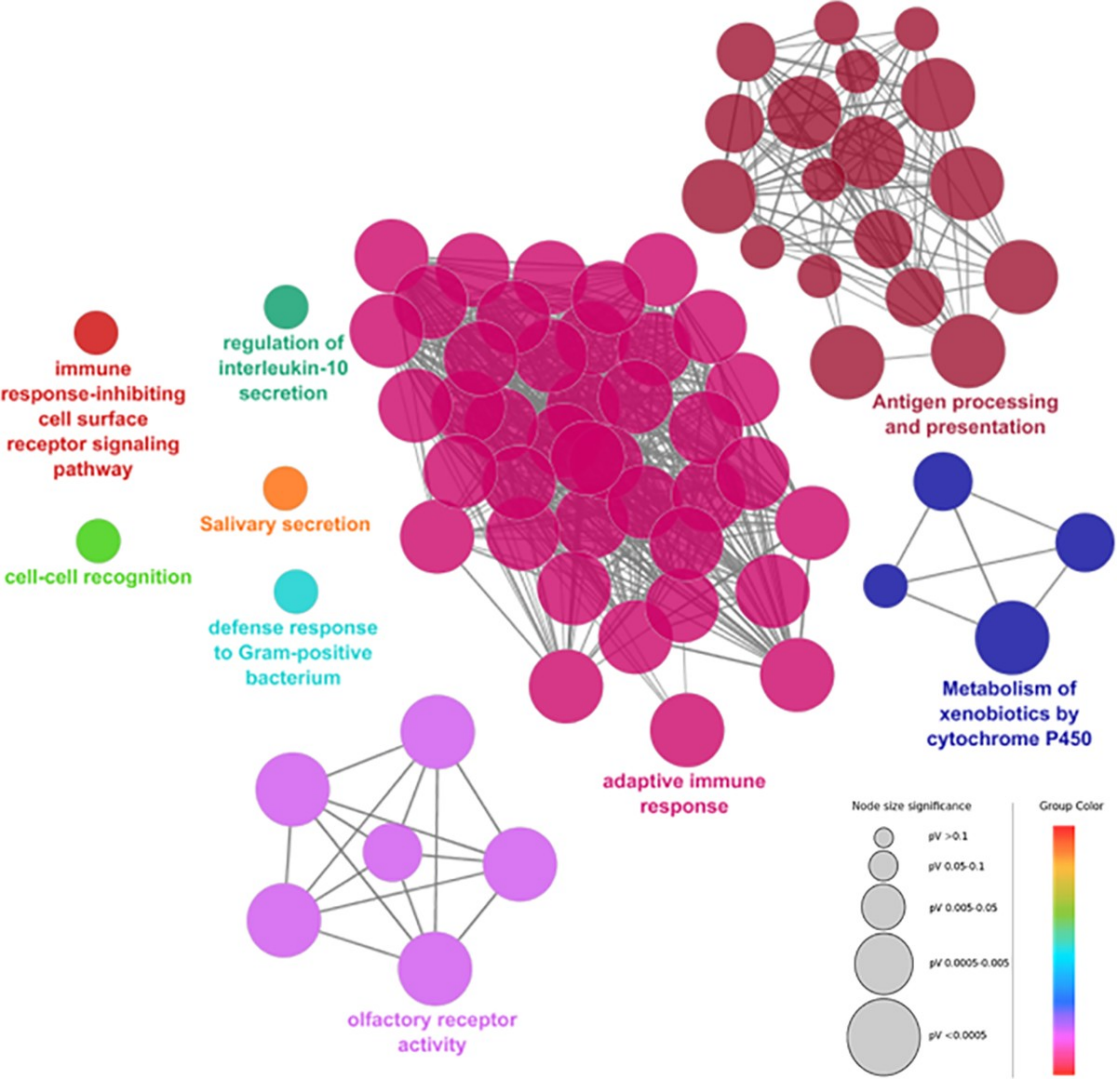
Enrichment network of the biological process Gene Ontology (GO) terms, Wikipathways and KEGG pathways identified by the functional annotation analysis using ClueGO cytoscape plug-in [34]. GO terms and pathways are grouped based on their biological role. The most significant term of a group is considered to be the leading term and it is highlighted in the network. The node size is proportional to the enrichment significance (only significantly enriched GO terms/pathways are visualized <0.05) and the node color reflects the functional group to which it belongs.

Gene set enrichment analysis was performed based on biological process GO terms, Wikipathways and KEGG pathways to explore the main functions of the genes disrupted by CNVs in breast cancer patients. The top 10 enriched GO terms and pathways are illustrated in S2 Table. The obtained results were visualized as a functionally organized network in order to group highly overlapping gene sets into functional clusters (Fig 2). Adaptive immune response, antigen processing and presentation, olfactory receptor activity and xenobiotics metabolism by cytochrome P450 were the main enriched functions. Interestingly, analysis of biological pathways supplied by Wikipathways revealed an enrichment of Tamoxifen metabolism (p-value = 0.01743 (Fisher exact test)).

### Rare copy number variations likely associated with malignancy

**Fig 3:**
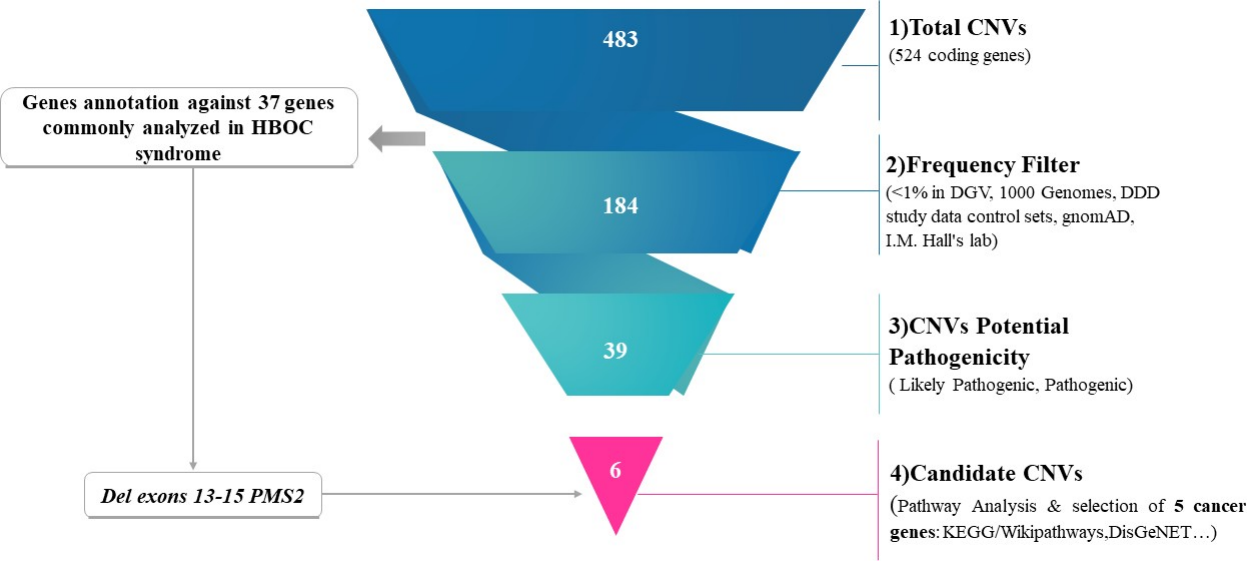
CNVs prioritization and identification of candidate CNVs. 1) Represents the total number of CNVs identified in breast cancer patients. 2)Elimination of CNVs with a frequency ≥1% in DGV,1000 genomes project, DDD study data control sets, gnomAD and I.M. Hall’s lab. 3)Selection of CNVs classified as likely pathogenic or pathogenic. 4)Functional features and biological pathways investigations and selection of CNVs disrupting cancer genes according to KEGG/wikipathways, OMIM expanded disease, DisGeNET.

In order to identify the most relevant CNVs that might be associated with hereditary breast cancer predisposition, we have first looked for deletions and duplications within 37 genes frequently analyzed in high risk breast and ovarian cancer families [41]. The full list of genes investigated is shown in S3 Table. Two unrelated patients originating from two distinct geographical regions (BC22 and BC37) carried a 20.8kb heterozygous deletion on 7p22.1 locus overlapping *RSPH10B* (exons 2–7) and *PMS2* (exons 13–15) genes. This CNV is not described in the DGV database yet it overlaps with a rare and pathogenic deletion reported in the dbVar database (nssv8639488). No additional deleterious CNVs have been identified on the remaining known genes. Therefore, we have applied several filters to detect CNVs on other genes that may contribute to disease susceptibility. Common CNVs with a frequency ≥1% have been eliminated. A total of 184 CNVs remained and were then filtered according to their potential pathogenicity. Only CNVs ranked as pathogenic or likely pathogenic were kept which reduces the number of CNVs to 39 (Fig 3). The remaining CNVs were further filtered to keep only those disrupting cancer genes. Five relevant CNVs have been identified as affecting the following cancer genes *APC2*, *POU5F1*, *KANSL1*, *DOCK8* and *TMTC3*. CNVs encompassing *DOCK8* and *KANSL1* were classified as pathogenic, while those identified on *APC2*, *POU5F1* and *TMTC3* were ranked as likely pathogenic (Table 2 and Fig 3).

**Table 2:** Candidates rare CNVs likely associated with malignancy.

Chr	Start	End	CNV length (pb)	CNV type	Genes	Affected exons	Affected Pathway (KEGG/Wikipathways)	DAVID Disease Class	Network of Cancer genes annotation	KEGG/OMIM expanded disease annotation	DGV Frequency	AnnotSV ranking	Patients
**6**	31083803	31133824	50021	DUP	*CDSN*, *PSORS1C2*, *PSORS1C1*, *CCHCR1*, *TCF19***, *POU5F1***	33	Wnt Signaling Pathway and Pluripotency (***POU5F1***)	Cancer	***(POU5F1)*** Oncogene	**-**	-	4^a^	BC39
**9**	214510	340321	125811	DUP	*C9orf66*, ***DOCK8***	14	Factors involved in megakaryocyte development and platelet production pathway *(****DOCK8)***	Hematological	(***DOCK8)*** Candidate cancer gene mutated in neuroblastomas [42]	(***DOCK8)*** Autosomal dominant mental retardation / Hyper-IgE syndrome	0.00322327	5^b^	BC40
**12**	88512261	88554006	41745	DUP	*CEP290*, ***TMTC3***	20	Pathways in clear cell renal cell carcinoma (*CEP290*)	-	*(****TMTC3)*** A candidate cancer gene mutated in Pancreatic cancer [43]	*(CEP290)* Meckel syndrome /Joubert syndrome /Leber congenital amaurosis	-	4	BC19
**17**	43515225	44159908	644683	DEL	*PLEKHM1*, *CRHR1*, *SPPL2C*, *MAPT*, *STH*, ***KANSL1***	53	MAPK Signaling Pathway (*MAPT)*	Cancer	(***KANSL1)*** A candidate cancer gene mutated in Bladder cancer and in multiple other cancers [44, 45]	Koolen-De Vries syndrome /*(CRHR1)* breast, prostate, lung, colorectal, pancreatic, ovarian endometrial cancers***/*** sarcoma***/*** melanoma/lymphoma	-	5	BC37
							Notch Signaling Pathway *(MAPT)*						
							Pathways Affected in Adenoid Cystic Carcinoma (***KANSL)***						
**17**	43515225	44249509	734284	DEL	*PLEKHM1*, *CRHR1*, *SPPL2C*, *MAPT*, *STH*, ***KANSL1***	55	MAPK Signaling Pathway (*MAPT)*	Cancer	(***KANSL1)*** A candidate cancer gene mutated in Bladder cancer and in multiple other cancers [44, 45]	Koolen-De Vries syndrome /*(CRHR1)* breast, prostate, lung, colorectal, pancreatic, ovarian endometrial cancers***/*** sarcoma***/*** melanoma/lymphoma	-	5	BC39
							Notch Signaling Pathway *(MAPT)*						
							Pathways Affected in Adenoid Cystic Carcinoma ***(KANSL1)***						
**19**	1461037	1483940	22903	DEL	***APC2***, *C19orf25*, *PCSK4*	11	***(APC2)*** *Wnt* signaling pathway	Pharmacogenomic	-	***(APC2)*** Colorectal cancer / Medulloblastoma	-	4	BC47
							Breast, Endometrial, Gastric and Colorectal pathways						
							Chromosomal and microsatellite instability in colorectal cancer						

Abbreviations: DUP: duplication, DEL: deletion

^a^: Likely Pathogenic

^b^: Pathogenic; genes in bold: selected cancer genes.

**Fig 4:**
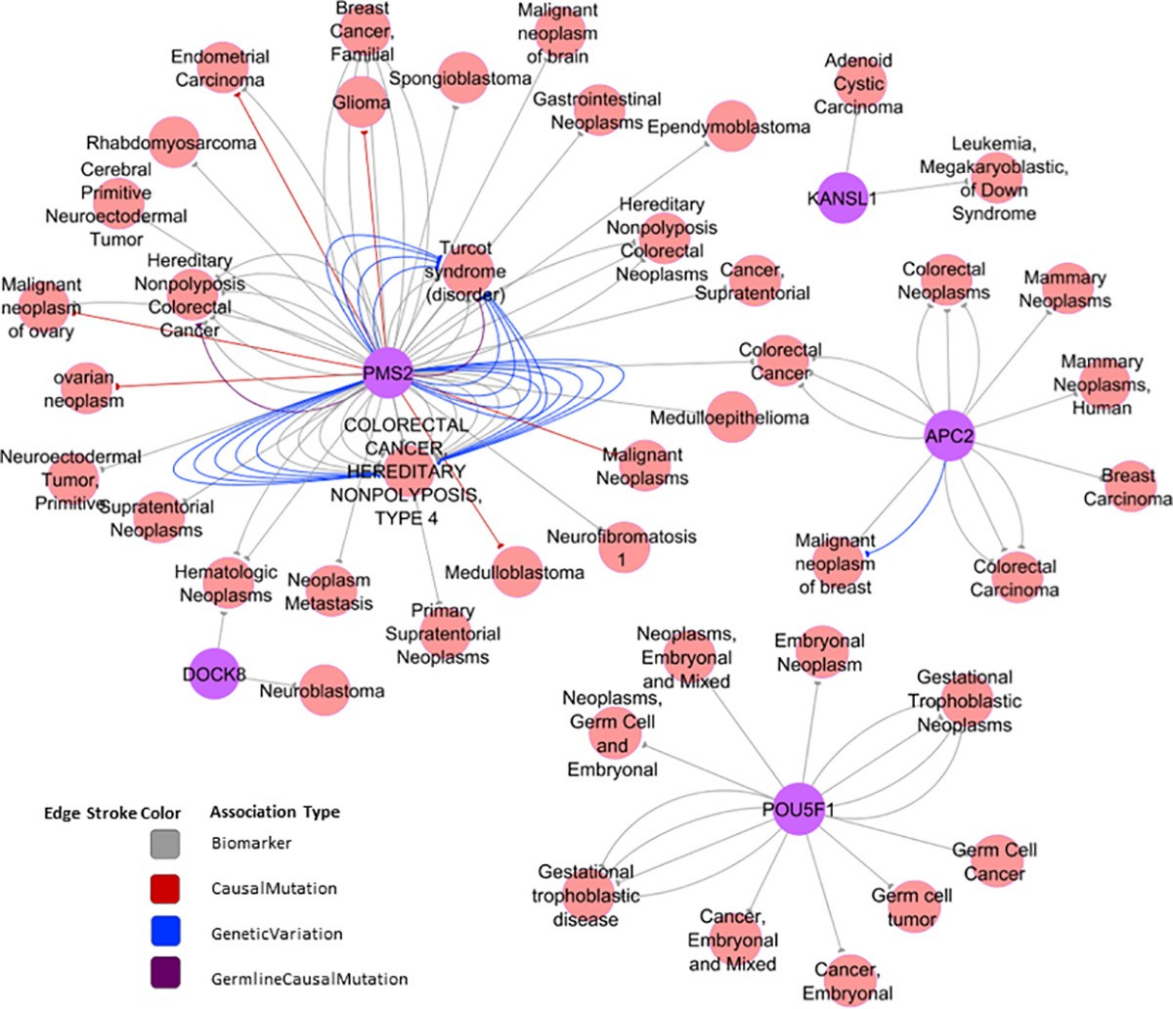
Gene disease association network of the candidate CNVs. The Gene Disease Association (GDA) network was generated by DisGeNET Cytoscape App [39] using curated data on neoplasms from all expert curated databases in DisGeNet. Each edge in the network represents the supporting evidence for a gene disease association uniquely defined by the source, one association type, and one publication. The colour of each edge distinguishes the association type.

Functional gene annotation, biological pathways investigations and gene disease network analysis (Fig 4) revealed relevant features for the five selected candidate CNVs. *KANSL1* gene which is mapped to pathways affected in adenoid cystic carcinoma was disrupted due to two large deletions of 644.6kb and 734.2kb identified in two unrelated patients, BC37 and BC39, respectively. Based on our analysis, this gene seems to be associated with adenoid cystic carcinoma and leukemia. Duplication in *DOCK8* was detected in one patient (BC40) and was found to be associated with neuroblastoma and hematologic neoplasms. The identification of CNVs within *APC2* (BC47) and *POU5F1* (BC39) was of interest as these genes were assigned to the Wnt signaling pathway which had a critical role in regulating cell proliferation and differentiation. Interrogation of KEGG disease and DiSgeNET databases revealed an association between *APC2* gene and colorectal cancer, medulloblastoma and breast cancer, while *POU5F1* was mainly associated with germ cell tumors. One patient (BC19) carried a duplication in *TMTC3* gene. According to the most recent update of the Network of Cancer Genes database, *TMTC3* is considered as a candidate cancer gene significantly mutated in pancreatic cancers with both point mutations and CNVs that have been detected. In two families (BC1 and BC52) CNVs prioritization did not reveal any potentially relevant rare CNVs. For family BC1, two related members have been sequenced and we focused our analysis only on rare CNVs shared between the two members to confirm the familial segregation. No rare CNVs have been detected in this family. This was the case also for BC52, suggesting that rare CNVs do not contribute to breast cancer susceptibility in these two families.

### Common copy number variations likely associated with breast cancer risk

To evaluate whether detected common CNVs overlap with CNVs known to be associated with breast cancer risk, a literature review has been conducted. Interestingly, several common CNVs identified in the current study are overlapping with CNV regions that were previously reported as associated with an increased risk of breast cancer at 1.28 to 2.9 folds (p-value = 0.02 to 1.10 × 10−06). This mainly involves the following 8 genes: *UGT2B15*, *UGT2B17*, *OR4C11*, *OR4P4*, *OR4S2*, *APOBEC3A*, *APOBEC3B* and *GSTT1* (Additional data are given in Table 3). These CNVs may contribute to breast cancer heritability through a polygenic risk model particularly for BC52. Indeed, this patient harbored several CNVs reported as associated with breast cancer risk involving *UGT2B17*, *OR4C11*, *OR4P4*, *OR4S2* and *GSTT1* genes. In addition, a homozygous deletion of *UGT2B17* was also detected in BC22 and BC40 and heterozygous deletions encompassing *APOBEC3A/B* and *GSTT1* genes were detected in BC39 and BC40 respectively.

**Table 3:** Common copy number variations overlapping with CNVs region known to be associated with breast cancer risk.

Chr	Start	End	CNV length	CNV type	Genotype	Genes	Affected exons	Patient	Reference	OR (If available)	P-value
**4**	69340442	69434202	93760	DEL	Homozygous	*TMPRSS11E*, *UGT2B17*	11	BC52	[4, 19, 46]	2.2–2.9	6× 10–4; p < 0.0001
**4**	69337179	69536336	199157	DEL	Homozygous	*TMPRSS11E*, UGT2B17*, UGT2B15**	18	BC22	[4, 19, 46]	*2.2–2.9	*6× 10–4; p < 0.0001; **1.29 × 10−03 to 1.10 × 10−06
**4**	69403344	69434202	30858	DEL	Homozygous	*UGT2B17*	6	BC40	[4, 19, 46]	2.2–2.9	6× 10–4; p < 0.0001
**11**	55370918	55419315	48397	DEL	Heterozygous	*OR4C11*, *OR4P4*, *OR4S2*	3	BC52	[4, 19]	2.6; 2.4; 2.1	7× 10–6; 2× 10–5; 4× 10–4
**22**	39358501	39380236	21735	DEL	Heterozygous	*APOBEC3A*, *APOBEC3B*	3	BC39	[19, 47, 48]	1.28	< 0.001
**22**	39387338	39388450	1112	DEL	Heterozygous	*APOBEC3B*	3	BC39	[19, 47, 48]	1.28	< 0.001
**22**	24376424	24384231	7807	DEL	Homozygous	*GSTT1*	5	BC52	[49]	1.6	0,02
**22**	24324820	24384231	59411	DEL	Heterozygous	*GSTT2*, *GSTT1*	8	BC40	[49]	1.6	0,02

### CNVs in genes involved in tamoxifen metabolism and treatment outcome

Tamoxifen metabolism pathway was found to be enriched in breast cancer patients involving *UGT2B15*, *SULT1A1* and *CYP2D6* genes. CNVs in these genes might influence sensitivity to tamoxifen treatment. Based on available clinical data and taking into account the limited number of cases, we tried to assess the response to hormonal therapy of patients carriers of these CNVs. Indeed, BC22 carried deletions of *UGT2B15*, *SULT1A1* and duplication of *CYP2D6* genes while 3 other patients (BC37, BC40 and BC52) harbored deletions in *SULT1A1* gene. We observed that all these patients had a good clinical response to tamoxifen with absence of disease recurrence for at least 12 months from the beginning of the endocrine therapy (Table 1).

### Identification of copy number variable regions and estimation of their frequencies in the Tunisian population

**Fig 5:**
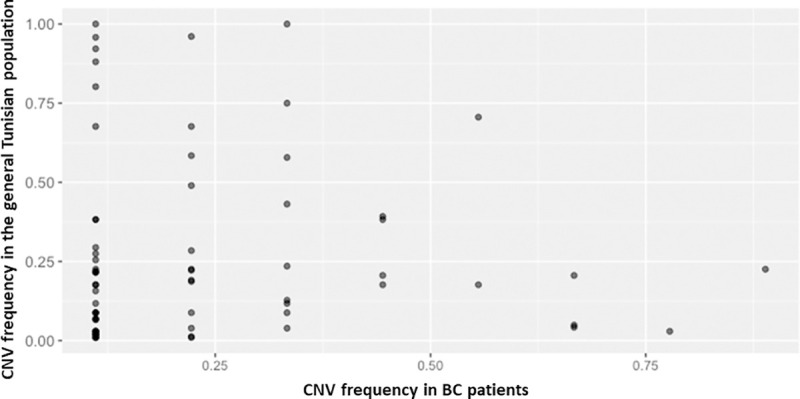
Frequencies of CNVs identified in breast cancer patients compared to those of the general Tunisian population. This figure illustrates the frequencies of the CNVs shared between breast cancer patients and the general Tunisian population.

In order to assess the accuracy of our data we have mapped our CNV calls to data from the study of (Romdhane et al,2020, under revision) (1083 CNVRs) identified in a cohort of 102 Tunisian individuals from the general population that were investigated using the Affymetrix Genome-Wide Human SNP Array 6.0. Prior to mapping we have first merged contiguous CNVs into a single Copy Number Variable Region (CNVR). This resulted in the detection of 280 CNVRs/CNVs (92 CNVRs with contiguous CNVs and 188 unique CNVs) consisting of 177 deletions, 67 duplications and 36 mixed loci constituted of both deletions and duplications. Frequencies of the CNVRs/CNVs identified in breast cancer patients compared to those of the Tunisian general population are illustrated in (Fig 5). Interestingly, 58 out of 280 (20%) of our CNVRs/CNVs overlapped with data reported in the Tunisian population. All shared CNVRs/CNVs were mapped to public data on structural variations from the DGV, the 1000 genomes project, the DDD study or the I.M. Hall's lab which provide confidence in the CNV calling method used in this study. The majority of these CNVRs/CNVs were common (having a frequency >1% in the public databases) and were found to affect enriched pathways such as olfactory receptor activity and xenobiotics metabolism. The remaining 222 CNVRs/CNVs were unique to breast cancer patients and are thought to contain CNVs associated with the disease susceptibility given their rarity in the Tunisian population. This was confirmed by our analysis since all candidate CNVs that we have identified and that were found to affect the cancer genes *PMS2*, *APC2*, *POU5F1*, *KANSL1*, *DOCK8* and *TMTC3* are part of this category.

## Discussion

The contribution of germline DNA copy number variations in breast cancer risk remains relatively undefined compared with the well documented association between point mutations and breast cancer susceptibility. Over the last decades, much advance has been made in the field of CNVs detection [50]. Nevertheless, the assessment of whether a CNV is benign or affects vital biological function is still challenging [50]. In the current study several CNVs were called and overrepresentation enrichment analysis showed an enrichment in immune response, olfactory receptor activity and xenobiotic metabolism functions and this is in agreement with what have been described in the CVN map of the human genome [5]. Moreover, the called CNVs were found to be unequally distributed among chromosomes. We have interestingly found a high proportion of copy number deletions within chromosome 17. Indeed, abnormalities affecting this chromosome are well recognized to play an important role in tumorigenesis and often arise in breast cancer. These aberrations include ERBB2 amplification, BRCA1 loss, P53 loss, and TOP2A amplification or deletion that are known to play important roles in breast cancer pathophysiology and treatment response [51, 52]. Subsequent analyses allowed the identification of several rare and common CNVs that may contribute to hereditary predisposition in patients who do not harbor pathogenic mutations in known breast cancer susceptibility genes. Six rare CNVs were believed to be the most relevant. Of special interest, was a rare pathogenic copy number deletion in the mismatch repair (MMR) gene *PMS2* involving exons 13–15 deletion that was detected in two unrelated patients. Mutations in *PMS2* are linked to Lynch syndrome, which is characterized by early incidence of colorectal cancer, along with increased risk of other malignancies including endometrial, ovarian, small bowel, and brain carcinoma. This same pathogenic deletion was previously identified in two patients with transverse colon cancer [53]. In the current report, none of the two breast cancer patients had personal or family history of the traditional malignancies associated with the Lynch syndrome. A recent research study showed that women with alterations in *PMS2* gene have a 3-fold increased risk for breast cancer and 37.7% cumulative risk by the age of 60 [54]. In the same study, it was shown that 11.1% of women with a Lynch syndrome alteration had no personal or family history of colorectal, endometrial, or ovarian cancer. Our findings along with those of the latter study suggest that women whose personal or family history is limited to breast cancer might carry *PMS2* alterations. It was also reported that patients with germline mutations in MMR genes are candidates for immunotherapy with PD-1 inhibitors regardless of cancer type [55]. Thus, the screening of the MMR repair genes alterations in breast cancer patients negative for *BRCA* mutations is recommended since it may help to select those who are candidates for immunotherapy, especially cases with metastatic or triple negative breast cancer. We have also identified a large deletion in 17q.21 locus spanning 6 genes *PLEKHM1*, *CRHR1*, *SPPL2C*, *MAPT*, *STH* and *KANSL1* in two unrelated patients. This CNV overlapped with known pathogenic deletion associated with Koolen–de Vries syndrome. This rare genetic disorder is clinically heterogenous characterized by developmental delay, moderate intellectual disability, distinctive facial features, and friendly demeanor. The clinical phenotype of this rare disorder is mainly linked to *KANSL1* gene deletion [56]. In the present study none of our two patients expressed the phenotype characterizing this disorder. However, mutations in *KANSL1* gene were also detected in multiple cancers [45] including bladder cancer [44] and germline copy number variation in this gene was also described in early colorectal cancer [57]. Moreover, among genes affected by this CNV, *PLEKHM1* (which is not deleted in Koolen–de Vries syndrome) is also considered as an ovarian cancer predisposing gene [58]. All these findings support the implication of *KANSL1* and *PLEKHM1* in cancer which may explain the phenotype of our two patients. In addition, other interesting genes were identified including *APC2* and *POU5F1*. These genes are mapped to the Wnt signaling pathway which has been highly associated with cancer [59]. This pathway is activated in a large fraction of breast cancers which contributes to tumor recurrence and lower overall survival. Indeed, this pathway also has implications for therapeutic interventions in cancers [60]. Taking the example of *POU5F1* gene, previous studies showed that the expression of this gene is required for the maintenance of transformed breast cancer cells and suggested its utility as a novel clinical biomarker and a potential target for gene-specific therapy of breast cancer [61]. In addition, alterations in *APC2* through loss of heterozygosity, promoter hypermethylation and somatic copy number aberrations were also described in breast tumors [62]. Moreover, we have identified a duplication in *DOCK8* gene that overlapped with a pathogenic CNV previously reported in individuals with developmental disabilities [63]. In addition to this, other reports suggested that *DOCK8* may have tumor suppressor functions. In fact, copy number deletions in this gene were described in human cancer particularly in neuroblastomas [42], in primary lung cancers, gastric and breast cancer cell lines [64]. Furthermore, one patient harbored a duplication in *TMTC3* gene which was found to be unregulated in breast cancer associated blood vessels and may therefore constitute a potentially anti-angiogenic target for breast cancer therapy [65]. CNVs in this gene were also detected in pancreatic cancers [43]. For one family (BC1), CNVs prioritization did not allow the identification of candidate rare CNVs potentially associated with breast cancer risk. Breast cancer susceptibility in this family is likely due to family specific genetic variants [25]. In the present study, several common CNVs overlapping with CNV regions previously reported as associated with breast cancer risk were identified including CNVs affecting *UGT2B15*, *UGT2B17*, *OR4C11*, *OR4P4*, *OR4S2*, *APOBEC3A*, *APOBEC3B* and *GSTT1* genes [4, 19]. Several studies have found an association between *APOBEC3* deletion and the risk of various cancers, particularly breast cancer with up to 1.3-fold increased risk. This locus was shown to be significantly associated with breast cancer risk in different populations including those of Chinese, Iranian, and European ancestries [18, 47, 48]. It was demonstrated also that deletion in the *APOBEC3* loci disrupting *APOBEC3A* and *APOBEC3B* genes lead to the decreased expression of the corresponding genes [66, 67]. Moreover, the association between *GSTT1* gene deletion and breast cancer risk has been widely studied and it was demonstrated that *GSTT1* null genotype is associated with increased breast cancer risk [68] and also with significant downregulation of *GSTT1* gene resulting in loss of protein expression [69–71]. This latter contributes to tumor cell survival by detoxification of numerous products induced by cancer therapy such as chemotherapy [49]. Interestingly *GSTT1* was previously investigated in the Tunisian population and results have shown significant association between the gene deletion and the risk of early onset of breast carcinoma [49]. On the other hand, the absence of *GSTT1* gene deletion was found to be significantly associated with poor clinical response to chemotherapy [49]. In the present study, response to chemotherapy cannot be effectively assessed due to the limited number of cases and since all patients received adjuvant treatment. Nevertheless, it is noteworthy that patients with *GSTT1* gene deletion (BC40, BC52) had a good survival with absence of cancer recurrence for at least 5 years, while disease relapse was observed in 3 patients (BC1-1, BC22, BC47) with a normal copy of *GSTT1*. In addition, two recently published reports showed that *OR4C11*, *OR4P4*, *OR4S2* and *UGT2B17* are associated with breast cancer with respectively 2.6, 2.4, 2.1 and 2.2-fold increase in breast cancer risk [4, 19] and it was proven also that the expression of *UGT2B17* gene is correlated with the corresponding germline CNVs [19]. Based on these observations we have suggested a polygenic inheritance for one patient as she harbored CNVs in all the above genes. The assessment of whether these CNVs could be associated with breast cancer risk in the Tunisian population will be of keen interest and need to be conducted in a larger cohort. In addition, our pathway analysis resulted in mapping some common CNVs namely *CYP2D6*, *UGT2B15* and *SULT1A1* to tamoxifen metabolism. In the present report, *SULT1A1* and *UGT2B15* deletions seem to correlate with good clinical response to tamoxifen. In fact, tamoxifen and its metabolites are inactivated by these genes through sulfation and glucuronidation respectively. It has been demonstrated that *SULT1A1* copy number is highly associated with the enzymatic activity, which is considered as a predictive biomarker for tamoxifen response [72]. A duplication within *CYP2D6* was detected in one patient receiving tamoxifen treatment. This gene catalyzes the transformation of the tamoxifen to its active form 4-OH-TAM [73] and it was suggested that a subject with duplication of active *CYP2D6* will metabolize drugs at an ultra-rapid rate, which could lead to a loss of therapeutic efficacy at standard doses [74]. Contrarily, in the present study, the patient carrying *CYP2D6* gene duplication had a good clinical response to tamoxifen therapy. The evaluation of the clinical relevance of CNVs in tamoxifen-metabolizing genes to drug efficacy in Tunisian breast cancer patients is of important interest since it may help to improve therapeutic decisions.

Here we described a substantial number of CNVs that might be of clinical interest in Tunisian breast cancer patients using WES data. This report is the first to use WES in the analysis of CNVs in Tunisian BRCAx families and it is considered to be among the first studies to elucidate the contribution of CNVs to disease susceptibility in BRCA negative families using WES data. Nonetheless, the findings of this study have to be seen in light of some limitations mainly related to the small sample size investigated. This could be explained in part by the rarity of BRCA negative familial breast cancer cases especially that the incidence of breast cancer in Tunisia is lower compared to that in developed countries and also by the limited resources that hampers the generation of an important number of exomes. Nevertheless, it is important to note that exome sequencing has previously been shown to be a valuable tool for detecting germline CNVs. Indeed, integration of CNV analysis in exome sequencing data-analysis pipelines, which until now have mostly focused on single nucleotide variants analysis, seems to be a promising approach for the detection of most of the alterations associated with disease susceptibility in a cost-effective manner. However, the specificity and the number of CNVs identified vary greatly depending on the used platforms and the CNVs detection algorithms [75]. In fact, benchmarking of several CNVs detection tools from exome data showed that a significant fraction of called CNVs are only present in a single tool [76]. It was demonstrated also that ExomeDepth is one the most balanced tools concerning sensitivity and specificity [77] and this latter was supported to be integrated with routine targeted NGS diagnostic services for Mendelian diseases [78]. Additionally, clinically relevant CNVs resulting from the different breast cancer studies highly depend on the bioinformatic tools and the methodology used to prioritize variants and to interpret results. To overcome these challenges, it is important to perform large scale studies, to pool data from previous reports, to analyze CNVs by combining different algorithms and to interpret the called CNVs using a consistent approach.

## Conclusions

In this study, we have identified a number of germline CNVs that possibly increase the susceptibility to breast cancer and that could therefore explain a fraction of familial breast cancer cases particularly those with no mutations in the major susceptibility genes. Screening of CNVs found in Wnt and MMR pathways must be considered in breast cancer patients since it might help to guide personalized therapeutic decisions. Furthermore and taking into account the genetic proximity with other populations in Middle East and North Africa (MENA) region, the present study will have an impact on molecular diagnosis of breast cancer not only for Tunisian patients but also for patients from other neighboring countries.

## Supporting information

S1 Table Coverage statistics and summary of CNVs data among breast cancer patients. (DOCX)

S2 Table Top 10 enriched biological process GO terms and pathways revealed by gene set enrichment analysis. (DOCX)

S3 Table Known cancer predisposition genes frequently investigated in hereditary breast and ovarian cancer families. (DOCX)

## References

[1] Bray F, Ferlay J, Soerjomataram I, Siegel RL, Torre LA, Jemal A. Global cancer statistics 2018: GLOBOCAN estimates of incidence and mortality worldwide for 36 cancers in 185 countries. CA: a cancer journal for clinicians. 2018;68(6):394–424. 30207593 10.3322/caac.21492

[2] Economopoulou P, Dimitriadis G, Psyrri A. Beyond BRCA: new hereditary breast cancer susceptibility genes. Cancer Treat Rev. 2015;41(1):1–8. Epub 2014/12/04. 10.1016/j.ctrv.2014.10.008 . 25467110

[3] Masson AL, Talseth-Palmer BA, Evans T-J, Grice DM, Hannan GN, Scott RJ. Expanding the genetic basis of copy number variation in familial breast cancer. Hereditary cancer in clinical practice. 2014;12(1):15 10.1186/1897-4287-12-15 24955146 PMC4064283

[4] Walker LC, Pearson JF, Wiggins GA, Giles GG, Hopper JL, Southey MC. Increased genomic burden of germline copy number variants is associated with early onset breast cancer: Australian breast cancer family registry. Breast Cancer Res. 2017;19(1):30 10.1186/s13058-017-0825-6 28302160 PMC5356248

[5] Zarrei M, MacDonald JR, Merico D, Scherer SW. A copy number variation map of the human genome. Nat Rev Genet. 2015;16(3):172–83. 10.1038/nrg3871 . 25645873

[6] Schmidt AY, Hansen TVO, Ahlborn LB, Jonson L, Yde CW, Nielsen FC. Next-Generation Sequencing-Based Detection of Germline Copy Number Variations in BRCA1/BRCA2: Validation of a One-Step Diagnostic Workflow. J Mol Diagn. 2017;19(6):809–16. 10.1016/j.jmoldx.2017.07.003 . 28822785

[7] Krepischi ACV, Pearson PL, Rosenberg C. Germline copy number variations and cancer predisposition. Future oncology. 2012;8(4):441–50. 10.2217/fon.12.34 22515447

[8] Krepischi AC, Achatz MI, Santos EM, Costa SS, Lisboa BC, Brentani H, et al Germline DNA copy number variation in familial and early-onset breast cancer. Breast Cancer Res. 2012;14(1):R24 10.1186/bcr3109 22314128 PMC3496142

[9] Kwong A, Chen J, Shin VY, Ho JC, Law FB, Au CH, et al The importance of analysis of long-range rearrangement of BRCA1 and BRCA2 in genetic diagnosis of familial breast cancer. Cancer Genet. 2015;208(9):448–54. 10.1016/j.cancergen.2015.05.031 . 26271414

[10] Nunziato M, Starnone F, Lombardo B, Pensabene M, Condello C, Verdesca F, et al Fast Detection of a BRCA2 Large Genomic Duplication by Next Generation Sequencing as a Single Procedure: A Case Report. Int J Mol Sci. 2017;18(11). 10.3390/ijms18112487 29165356 PMC5713453

[11] Germani A, Libi F, Maggi S, Stanzani G, Lombardi A, Pellegrini P, et al Rapid detection of copy number variations and point mutations in BRCA1/2 genes using a single workflow by ion semiconductor sequencing pipeline. Oncotarget. 2018;9(72):33648 10.18632/oncotarget.26000 30263092 PMC6154752

[12] Riahi A, Chabouni-Bouhamed H, Kharrat M. Prevalence of BRCA1 and BRCA2 large genomic rearrangements in Tunisian high risk breast/ovarian cancer families: Implications for genetic testing. Cancer genetics. 2017;210:22–7. 10.1016/j.cancergen.2016.11.002 28212807

[13] Tchatchou S, Burwinkel B. Chromosome copy number variation and breast cancer risk. Cytogenet Genome Res. 2008;123(1–4):183–7. 10.1159/000184707 . 19287154

[14] Pylkas K, Vuorela M, Otsukka M, Kallioniemi A, Jukkola-Vuorinen A, Winqvist R. Rare copy number variants observed in hereditary breast cancer cases disrupt genes in estrogen signaling and TP53 tumor suppression network. PLoS Genet. 2012;8(6):e1002734 10.1371/journal.pgen.1002734 22737080 PMC3380845

[15] Kuusisto KM, Akinrinade O, Vihinen M, Kankuri-Tammilehto M, Laasanen SL, Schleutker J. copy number variation analysis in familial BRCA1/2-negative Finnish breast and ovarian cancer. PLoS One. 2013;8(8):e71802 10.1371/journal.pone.0071802 23967248 PMC3742470

[16] Shlien A, Malkin D. Copy number variations and cancer. Genome Med. 2009;1(6):62 10.1186/gm62 19566914 PMC2703871

[17] Long J, Delahanty RJ, Li G, Gao YT, Lu W, Cai Q, et al A common deletion in the APOBEC3 genes and breast cancer risk. J Natl Cancer Inst. 2013;105(8):573–9. 10.1093/jnci/djt018 23411593 PMC3627644

[18] Xuan D, Li G, Cai Q, Deming-Halverson S, Shrubsole MJ, Shu X-O, et al APOBEC3 deletion polymorphism is associated with breast cancer risk among women of European ancestry. Carcinogenesis. 2013;34(10):2240–3. 10.1093/carcin/bgt185 23715497 PMC3786378

[19] Kumaran M, Cass CE, Graham K, Mackey JR, Hubaux R, Lam W, et al Germline copy number variations are associated with breast cancer risk and prognosis. Sci Rep. 2017;7(1):14621 10.1038/s41598-017-14799-7 29116104 PMC5677082

[20] Hu XY, Huang XY, Ma J, Zuo Y, Luo NB, Lai SL, et al GSTT1 and GSTM1 polymorphisms predict treatment outcome for breast cancer: a systematic review and meta-analysis. Tumour Biol. 2016;37(1):151–62. 10.1007/s13277-015-4401-3 . 26577857

[21] Saladores PH, Precht JC, Schroth W, Brauch H, Schwab M. Impact of metabolizing enzymes on drug response of endocrine therapy in breast cancer. Expert review of molecular diagnostics. 2013;13(4):349–65. 10.1586/erm.13.26 . 23638818

[22] Yang J, Shen J, Tang S, Zhou Y, Song W, Zhou Y, et al The outcomes of tamoxifen therapy in breast cancer patients and genotypes of SULT1A1 and glucuronosyltransferase. Int J Clin Exp Med. 2017;10(4):6273–82.

[23] Magi A, Tattini L, Pippucci T, Torricelli F, Benelli M. Read count approach for DNA copy number variants detection. Bioinformatics. 2011;28(4):470–8. 10.1093/bioinformatics/btr707 22199393

[24] Miller S, Dykes D, Polesky H. A simple salting out procedure for extracting DNA from human nucleated cells. Nucleic acids research. 1988;16(3):1215 10.1093/nar/16.3.1215 3344216 PMC334765

[25] Hamdi Y, Boujemaa M, Ben Rekaya M, Ben Hamda C, Mighri N, El Benna H, et al Family specific genetic predisposition to breast cancer: results from Tunisian whole exome sequenced breast cancer cases. J Transl Med. 2018;16(1):158 10.1186/s12967-018-1504-9 . 29879995 PMC5992876

[26] Van der Auwera GA, Carneiro MO, Hartl C, Poplin R, Del Angel G, Levy‐Moonshine A, et al From FastQ data to high‐confidence variant calls: the genome analysis toolkit best practices pipeline. Current protocols in bioinformatics. 2013;43(1):11.0. 1–.0. 33. 10.1002/0471250953.bi1110s43 25431634 PMC4243306

[27] Desvignes J-P, Bartoli M, Delague V, Krahn M, Miltgen M, Béroud C, et al VarAFT: a variant annotation and filtration system for human next generation sequencing data. Nucleic acids research. 2018 10.1093/nar/gky471 29860484 PMC6030844

[28] Plagnol V, Curtis J, Epstein M, Mok KY, Stebbings E, Grigoriadou S, et al A robust model for read count data in exome sequencing experiments and implications for copy number variant calling. Bioinformatics. 2012;28(21):2747–54. 10.1093/bioinformatics/bts526 22942019 PMC3476336

[29] ronique Geoffroy V, Herenger Y, Kress A, Muller J. AnnotSV: An integrated tool for Structural Variations annotation. Bioinformatics. 2018;1:3 10.1093/bioinformatics/bty304 29669011

[30] MacDonald JR, Ziman R, Yuen RK, Feuk L, Scherer SW. The Database of Genomic Variants: a curated collection of structural variation in the human genome. Nucleic Acids Res. 2014;42(Database issue):D986–92. 10.1093/nar/gkt958 24174537 PMC3965079

[31] Clarke L, Zheng-Bradley X, Smith R, Kulesha E, Xiao C, Toneva I, et al The 1000 Genomes Project: data management and community access. Nature methods. 2012;9(5):459 10.1038/nmeth.1974 22543379 PMC3340611

[32] Abel HJ, Larson DE, Regier AA, Chiang C, Das I, Kanchi KL, et al Mapping and characterization of structural variation in 17,795 human genomes. Nature. 2020;583(7814):83–9. 10.1038/s41586-020-2371-0 32460305 PMC7547914

[33] Kuleshov MV, Jones MR, Rouillard AD, Fernandez NF, Duan Q, Wang Z, et al Enrichr: a comprehensive gene set enrichment analysis web server 2016 update. Nucleic acids research. 2016;44(W1):W90–W7. 10.1093/nar/gkw377 27141961 PMC4987924

[34] Bindea G, Mlecnik B, Hackl H, Charoentong P, Tosolini M, Kirilovsky A, et al ClueGO: a Cytoscape plug-in to decipher functionally grouped gene ontology and pathway annotation networks. Bioinformatics. 2009;25(8):1091–3. 10.1093/bioinformatics/btp101 19237447 PMC2666812

[35] An O, Pendino V, D'Antonio M, Ratti E, Gentilini M, Ciccarelli FD. NCG 4.0: the network of cancer genes in the era of massive mutational screenings of cancer genomes. Database (Oxford). 2014;2014:bau015 10.1093/database/bau015 24608173 PMC3948431

[36] Duncan D, Prodduturi N, Zhang B. WebGestalt2: an updated and expanded version of the Web-based Gene Set Analysis Toolkit. Bmc Bioinformatics. 2010;11(S4):P10.

[37] Kanehisa M, Furumichi M, Tanabe M, Sato Y, Morishima K. KEGG: new perspectives on genomes, pathways, diseases and drugs. Nucleic Acids Res. 2017;45(D1):D353–D61. 10.1093/nar/gkw1092 27899662 PMC5210567

[38] Jiao X, Sherman BT, Huang da W, Stephens R, Baseler MW, Lane HC, et al DAVID-WS: a stateful web service to facilitate gene/protein list analysis. Bioinformatics. 2012;28(13):1805–6. 10.1093/bioinformatics/bts251 22543366 PMC3381967

[39] Piñero J, Bravo À, Queralt-Rosinach N, Gutiérrez-Sacristán A, Deu-Pons J, Centeno E, et al DisGeNET: a comprehensive platform integrating information on human disease-associated genes and variants. Nucleic acids research. 2016:gkw943 10.1093/nar/gkw943 27924018 PMC5210640

[40] Gu Z, Gu L, Eils R, Schlesner M, Brors B. circlize implements and enhances circular visualization in R. Bioinformatics. 2014;30(19):2811–2. 10.1093/bioinformatics/btu393 24930139

[41] Suszynska M, Klonowska K, Jasinska AJ, Kozlowski P. Large-scale meta-analysis of mutations identified in panels of breast/ovarian cancer-related genes—Providing evidence of cancer predisposition genes. Gynecol Oncol. 2019;153(2):452–62. 10.1016/j.ygyno.2019.01.027 . 30733081

[42] Schramm A, Köster J, Assenov Y, Althoff K, Peifer M, Mahlow E, et al Mutational dynamics between primary and relapse neuroblastomas. Nature genetics. 2015;47(8):872 10.1038/ng.3349 26121086

[43] Wang L, Tsutsumi S, Kawaguchi T, Nagasaki K, Tatsuno K, Yamamoto S, et al Whole-exome sequencing of human pancreatic cancers and characterization of genomic instability caused by MLH1 haploinsufficiency and complete deficiency. Genome research. 2012;22(2):208–19. 10.1101/gr.123109.111 22156295 PMC3266029

[44] Robertson AG, Kim J, Al-Ahmadie H, Bellmunt J, Guo G, Cherniack AD, et al Comprehensive molecular characterization of muscle-invasive bladder cancer. Cell. 2017;171(3):540–56. e25. 10.1016/j.cell.2017.09.007 28988769 PMC5687509

[45] Bailey MH, Tokheim C, Porta-Pardo E, Sengupta S, Bertrand D, Weerasinghe A, et al Comprehensive characterization of cancer driver genes and mutations. Cell. 2018;173(2):371–85. e18. 10.1016/j.cell.2018.02.060 29625053 PMC6029450

[46] Eskandari-Nasab E, Hashemi M, Rezaei H, Fazaeli A, Mashhadi MA, Moghaddam SS, et al Evaluation of UDP-glucuronosyltransferase 2B17 (UGT2B17) and dihydrofolate reductase (DHFR) genes deletion and the expression level of NGX6 mRNA in breast cancer. Molecular biology reports. 2012;39(12):10531–9. 10.1007/s11033-012-1938-8 23053953

[47] Han Y, Qi Q, He Q, Sun M, Wang S, Zhou G, et al APOBEC3 deletion increases the risk of breast cancer: a meta-analysis. Oncotarget. 2016;7(46):74979 10.18632/oncotarget.11792 27602762 PMC5342716

[48] Rezaei M, Hashemi M, Hashemi SM, Mashhadi MA, Taheri M. APOBEC3 deletion is associated with breast cancer risk in a sample of southeast Iranian population. International journal of molecular and cellular medicine. 2015;4(2):103 26261799 PMC4499572

[49] Khedhaier A, Remadi S, Corbex M, Ahmed SB, Bouaouina N, Mestiri S, et al Glutathione S-transferases (GSTT1 and GSTM1) gene deletions in Tunisians: susceptibility and prognostic implications in breast carcinoma. Br J Cancer. 2003;89(8):1502–7. 10.1038/sj.bjc.6601292 14562023 PMC2394332

[50] Nowakowska B. Clinical interpretation of copy number variants in the human genome. Journal of applied genetics. 2017;58(4):449–57. 10.1007/s13353-017-0407-4 28963714 PMC5655614

[51] Vos CB, ter Haar NT, Rosenberg C, Peterse JL, Cleton-Jansen AM, Cornelisse CJ, et al Genetic alterations on chromosome 16 and 17 are important features of ductal carcinoma in situ of the breast and are associated with histologic type. Br J Cancer. 1999;81(8):1410–8. 10.1038/sj.bjc.6693372 10604741 PMC2362977

[52] Reinholz MM, Bruzek AK, Visscher DW, Lingle WL, Schroeder MJ, Perez EA, et al Breast cancer and aneusomy 17: implications for carcinogenesis and therapeutic response. The Lancet Oncology. 2009;10(3):267–77. 10.1016/S1470-2045(09)70063-4 19261255 PMC5549275

[53] Vaughn CP, Hart KJ, Samowitz WS, Swensen JJ. Avoidance of pseudogene interference in the detection of 3' deletions in PMS2. Hum Mutat. 2011;32(9):1063–71. 10.1002/humu.21540 . 21618646

[54] Roberts ME, Jackson SA, Susswein LR, Zeinomar N, Ma X, Marshall ML, et al MSH6 and PMS2 germ-line pathogenic variants implicated in Lynch syndrome are associated with breast cancer. Genet Med. 2018;20(10):1167–74. 10.1038/gim.2017.254 29345684 PMC6051923

[55] Le DT, Durham JN, Smith KN, Wang H, Bartlett BR, Aulakh LK, et al Mismatch repair deficiency predicts response of solid tumors to PD-1 blockade. Science. 2017;357(6349):409–13. 10.1126/science.aan6733 28596308 PMC5576142

[56] Koolen DA, Pfundt R, Linda K, Beunders G, Veenstra-Knol HE, Conta JH, et al The Koolen-de Vries syndrome: a phenotypic comparison of patients with a 17q21. 31 microdeletion versus a KANSL1 sequence variant. European Journal of Human Genetics. 2016;24(5):652 10.1038/ejhg.2015.178 26306646 PMC4930086

[57] Disciglio V, Devecchi A, Palumbo O, Carella M, Penso D, Milione M, et al Whole exome sequencing and single nucleotide polymorphism array analyses to identify germline alterations in genes associated with testosterone metabolism in a patient with androgen insensitivity syndrome and early-onset colorectal cancer. Chinese journal of cancer. 2016;35(1):51 10.1186/s40880-016-0115-1 27267075 PMC4897824

[58] Permuth-Wey J, Lawrenson K, Shen HC, Velkova A, Tyrer JP, Chen Z, et al Identification and molecular characterization of a new ovarian cancer susceptibility locus at 17q21. 31. Nature communications. 2013;4:1627 10.1038/ncomms2613 23535648 PMC3709460

[59] Zhan T, Rindtorff N, Boutros M. Wnt signaling in cancer. Oncogene. 2017;36(11):1461–73. 10.1038/onc.2016.304 27617575 PMC5357762

[60] Krishnamurthy N, Kurzrock R. Targeting the Wnt/beta-catenin pathway in cancer: Update on effectors and inhibitors. Cancer treatment reviews. 2018;62:50–60. 10.1016/j.ctrv.2017.11.002 29169144 PMC5745276

[61] Zhao F-Q, Misra Y, Li D-B, Wadsworth MP, Krag D, Weaver D, et al Differential expression of Oct3/4 in human breast cancer and normal tissues. International journal of oncology. 2018;52(6):2069–78. 10.3892/ijo.2018.4341 29620155

[62] Daly CS, Shaw P, Ordonez LD, Williams GT, Quist J, Grigoriadis A, et al Functional redundancy between Apc and Apc2 regulates tissue homeostasis and prevents tumorigenesis in murine mammary epithelium. Oncogene. 2017;36(13):1793–803. 10.1038/onc.2016.342 27694902 PMC5219933

[63] Miller DT, Adam MP, Aradhya S, Biesecker LG, Brothman AR, Carter NP, et al Consensus statement: chromosomal microarray is a first-tier clinical diagnostic test for individuals with developmental disabilities or congenital anomalies. The American Journal of Human Genetics. 2010;86(5):749–64. 10.1016/j.ajhg.2010.04.006 20466091 PMC2869000

[64] Zhang Q, Davis JC, Lamborn IT, Freeman AF, Jing H, Favreau AJ, et al Combined immunodeficiency associated with DOCK8 mutations. The New England journal of medicine. 2009;361(21):2046–55. 10.1056/NEJMoa0905506 19776401 PMC2965730

[65] Jones DT, Lechertier T, Mitter R, Herbert JM, Bicknell R, Jones JL, et al Gene expression analysis in human breast cancer associated blood vessels. PLoS One. 2012;7(10):e44294 10.1371/journal.pone.0044294 23056178 PMC3462779

[66] Klonowska K, Kluzniak W, Rusak B, Jakubowska A, Ratajska M, Krawczynska N, et al The 30 kb deletion in the APOBEC3 cluster decreases APOBEC3A and APOBEC3B expression and creates a transcriptionally active hybrid gene but does not associate with breast cancer in the European population. Oncotarget. 2017;8(44):76357 10.18632/oncotarget.19400 29100317 PMC5652711

[67] Pan J-W, Zabidi MMA, Chong B-K, Meng M-Y, Ng P-S, Hasan SN, et al Germline APOBEC3B deletion in Asian women increases somatic hypermutation in breast cancer that is associated with Her2 subtype, PIK3CA mutations, immune activation, and increased survival. bioRxiv. 2020. 10.1002/ijc.33463 33423300

[68] Sergentanis TN, Economopoulos KP. GSTT1 and GSTP1 polymorphisms and breast cancer risk: a meta-analysis. Breast cancer research and treatment. 2010;121(1):195–202. 10.1007/s10549-009-0520-0 19760040

[69] Curran JE, Weinstein SR, Griffiths LR. Polymorphisms of glutathione S-transferase genes (GSTM1, GSTP1 and GSTT1) and breast cancer susceptibility. Cancer Lett. 2000;153(1–2):113–20. 10.1016/s0304-3835(00)00361-x . 10779639

[70] Rotunno M, Lam TK, Vogt A, Bertazzi PA, Lubin JH, Caporaso NE, et al GSTM1 and GSTT1 copy numbers and mRNA expression in lung cancer. Molecular carcinogenesis. 2012;51(S1):E142–E50. 10.1002/mc.21890 22392686 PMC3376678

[71] Yang F, Xiong J, Jia X-E, Gu Z-H, Shi J-Y, Zhao Y, et al GSTT1 deletion is related to polycyclic aromatic hydrocarbons-induced DNA damage and lymphoma progression. PLoS One. 2014;9(2):e89302 10.1371/journal.pone.0089302 24586676 PMC3930712

[72] Moyer AM, Suman VJ, Weinshilboum RM, Avula R, Black JL, Safgren SL, et al SULT1A1,CYP2C19and disease-free survival in early breast cancer patients receiving tamoxifen. Pharmacogenomics. 2011;12(11):1535–43. 10.2217/pgs.11.97 21961651 PMC3235041

[73] Tengström M, Mannermaa A, Kosma V-M, Hirvonen A, Kataja V. SULT1A1 rs9282861 polymorphism-a potential modifier of efficacy of the systemic adjuvant therapy in breast cancer? BMC cancer. 2012;12(1):257 10.1186/1471-2407-12-257 22708928 PMC3388009

[74] Surekha D, Sailaja K, Rao DN, Padma T, Raghunadharao D, Vishnupriya S. CYP2D6* 4 polymorphisms and breast cancer risk. Biol Med. 2010;2:49–55.

[75] Pfundt R, Del Rosario M, Vissers L, Kwint MP, Janssen IM, de Leeuw N, et al Detection of clinically relevant copy-number variants by exome sequencing in a large cohort of genetic disorders. Genet Med. 2017;19(6):667–75. 10.1038/gim.2016.163 28574513 PMC5460076

[76] Tan R, Wang Y, Kleinstein SE, Liu Y, Zhu X, Guo H, et al An evaluation of copy number variation detection tools from whole‐exome sequencing data. Human mutation. 2014;35(7):899–907. 10.1002/humu.22537 24599517

[77] Moreno-Cabrera JM, Del Valle J, Castellanos E, Feliubadaló L, Pineda M, Brunet J, et al Benchmark of tools for CNV detection from NGS panel data in a genetic diagnostics context. BioRxiv. 2019:850958. 10.1038/s41431-020-0675-z PMC7784926 32561899

[78] Ellingford JM, Campbell C, Barton S, Bhaskar S, Gupta S, Taylor RL, et al Validation of copy number variation analysis for next-generation sequencing diagnostics. Eur J Hum Genet. 2017;25(6):719–24. 10.1038/ejhg.2017.42 28378820 PMC5427176

