# Supplementary material for: Germline copy number variations in BRCA1/2 negative families: Role in the molecular etiology of hereditary breast cancer in Tunisia
Source: PLoS One. 2021 Jan 27;16(1):e0245362. doi: 10.1371/journal.pone.0245362 (PMC7840007; doi:10.1371/journal.pone.0245362)
Supplement: S1 Table — (DOCX) [file pone.0245362.s001.docx]

***S1 Table. Coverage statistics and summary of CNVs data among breast cancer patients***

|  | **BC1** | | **BC19** | **BC22** | **BC37** | **BC39** | **BC40** | **BC47** | **BC52** |
| --- | --- | --- | --- | --- | --- | --- | --- | --- | --- |
|  | **BC1-1** | **BC1-8** |  |  |  |  |  |  |  |
| **Total reads** | 52 713 285 | 62 954 053 | 49 111 123 | 56 094 160 | 65 433 181 | 57 702 133 | 50 721 434 | 53 120 773 | 52 855 775 |
| **% Reads mapped to human genome** | 99.41 | 99.40 | 99.64 | 99.46 | 99.44 | 99.44 | 99.46 | 99.65 | 99.64 |
| **Total coverage** | 3 276 301 158 | 3 878 573 796 | 2 718 453 424 | 3 494 380 561 | 3 954 519 193 | 3 546 468 851 | 3 074 996 362 | 2 909 347 349 | 2 907 656 083 |
| **Mean read depth of target regions (X)** | 63.56 | 75.25 | 52.74 | 67.80 | 76.72 | 68.81 | 59.66 | 56.45 | 56.41 |
| **% Coverage of target regions (more than 10X)** | 91.9 | 93.0 | 91.3 | 92.3 | 93.0 | 92.6 | 91.7 | 91.7 | 91.6 |
| **% Coverage of target regions (more than 20X)**  ***High penetrance genes***  ***BRCA1***  ***BRCA2***  ***CDH1***  ***PTEN***  ***STK11***  ***TP53***  ***Moderate penetrance genes***  ***ATM***  ***CHEK2***  ***BRIP1***  ***PALB2*** | 85.3  100  99.51  96.72  100  95.08  92.64  97.86  81.6  99.39  100 | 88.0  100  99.38  98.19  100  98  97.72  96.67  80.69  99.6  100 | 82.7  99.8  99.44  96.19  98.43  91.78  98.35  95.53  82.06  95.52  100 | 92.3  100  99.5  97.62  99.92  98.23  94.25  97.67  81.2  99.49  100 | 88.1  100  99.51  96.3  98.68  97.08  99.49  98.08  85.24  100  100 | 86.9  100  99.39  97.17  100  96.47  99.07  97.6  82.34  97.79  100 | 84.6  99.71  98.82  96.45  100  97.7  90.86  95.65  82.06  98.11  99.83 | 83.9  99.84  99.35  95.28  100  91.24  90.69  96.48  82.79  99.01  99.89 | 83.8  100  97.95  96.6  100  96.16  99.11  93.71  79.78  97.81  100 |
| **Total CNVs (n)**  **Deletion**  **Duplication** | 64  46  18 | 82  54  28 | 51  30  21 | 77  51  26 | 86  54  32 | 86  63  23 | 93  50  43 | 56  43  13 | 76  52  24 |
| **Mean size of CNV (kb)** | 20.9 | 16.5 | 24.2 | 33.7 | 36.2 | 29.2 | 28.8 | 24.4 | 26 |
