# Supplementary material for: Germline copy number variations in BRCA1/2 negative families: Role in the molecular etiology of hereditary breast cancer in Tunisia
Source: PLoS One. 2021 Jan 27;16(1):e0245362. doi: 10.1371/journal.pone.0245362 (PMC7840007; doi:10.1371/journal.pone.0245362)
Supplement: S2 Table — (DOCX) [file pone.0245362.s002.docx]

***S2 Table. Top 10 enriched biological process GO terms and pathways revealed by Gene Set Enrichment analysis***

| **KEGG 2019 Human** | | | | | | |
| --- | --- | --- | --- | --- | --- | --- |
| **Index** | **Name** | **P-value** | **Adjusted p-value** | **Odds Ratio** | **Combined score** | **Genes** |
| **1** | **Antigen processing and presentation** | **5.673e-16** | **1.747e-13** | **10.25** | **359.93** | ***HLA-DRB5;KLRC2;KLRC3;KIR2DS4;HLA-B;RFXANK;KIR3DL1;HLA-A;KIR2DL1;KIR3DL2;HLA-F;KIR3DL3;HLA-G;KIR2DL3;KIR2DL4;HLA-DRA;KLRC1;HLA-DQA2;HLA-DQA1;HLA-DRB1;HLA-DQB1*** |
| **2** | **Graft-versus-host disease** | **6.517e-14** | **1.004e-11** | **13.75** | **417.59** | ***HLA-DRB5;HLA-B;KIR3DL1;KIR2DL1;KIR3DL2;HLA-A;HLA-F;KIR2DL3;HLA-G;HLA-DRA;KLRC1;HLA-DQA2;HLA-DQA1;HLA-DRB1;HLA-DQB1*** |
| **3** | **Type I diabetes mellitus** | **1.133e-8** | **0.000001163** | **9.62** | **175.96** | ***HLA-DRB5;HLA-B;HLA-DRA;HLA-A;HLA-F;HLA-DQA2;HLA-G;HLA-DQA1;HLA-DRB1;HSPD1;HLA-DQB1*** |
| **4** | **Allograft rejection** | **3.944e-8** | **0.000003037** | **9.89** | **168.66** | ***HLA-DRB5;HLA-B;HLA-DRA;HLA-A;HLA-F;HLA-DQA2;HLA-G;HLA-DQA1;HLA-DRB1;HLA-DQB1*** |
| **5** | **Autoimmune thyroid disease** | **0.000001137** | **0.00007004** | **7.09** | **97.09** | ***HLA-DRB5;HLA-B;HLA-DRA;HLA-A;HLA-F;HLA-G;HLA-DQA2;HLA-DQA1;HLA-DRB1;HLA-DQB1*** |
| **6** | **Natural killer cell mediated cytotoxicity** | **0.000002175** | **0.0001116** | **4.30** | **56.13** | ***KLRC2;SHC1;KLRC3;KIR2DS4;HLA-B;KIR3DL1;HLA-A;KIR2DL1;KIR3DL2;HLA-G;KIR2DL3;KIR2DL4;FCGR3B;KLRC1;MICA*** |
| **7** | **Viral myocarditis** | **0.000003175** | **0.0001397** | **6.37** | **80.67** | ***HLA-DRB5;HLA-B;HLA-DRA;HLA-A;HLA-F;HLA-G;HLA-DQA2;HLA-DQA1;HLA-DRB1;HLA-DQB1*** |
| **8** | **Staphylococcus aureus infection** | **0.00001186** | **0.0004567** | **5.53** | **62.71** | ***DEFB103A;HLA-DRB5;FCGR3B;HLA-DRA;DEFB4A;HLA-DQA2;FCGR2C;HLA-DQA1;HLA-DRB1;HLA-DQB1*** |
| **9** | **Olfactory transduction** | **0.00002458** | **0.0008411** | **2.37** | **25.16** | ***OR2A1;OR4K5;OR4K2;OR4K1;OR4M2;OR4M1;OR4Q3;OR2T34;OR2T35;OR2T10;OR4S2;OR2T11;OR2A42;OR1D5;OR2J3;OR4N4;OR2J2;OR2J1;OR4P4;OR5P3;OR4N2;OR5P2;OR52E8;OR4C11;OR51A4;OR2A7;OR51A2;OR2A4*** |
| **10** | **Metabolism of xenobiotics by cytochrome P450** | **0.00002541** | **0.0007828** | **5.08** | **53.75** | ***GSTM4;GSTM1;CYP2D6;UGT2B15;UGT2B17;UGT2B28;GSTT2;GSTT1;CYP2F1;GSTT2B*** |
| **Wikipathways** | | | | | | |
| **1** | **Allograft Rejection WP2328** | **0.00002385** | **0.01126** | **4.65** | **49.46** | ***HLA-DRB5;HLA-B;HLA-DRA;HLA-A;HLA-F;HLA-G;HLA-DQA2;MICA;HLA-DQA1;HLA-DRB1;HLA-DQB1*** |
| **2** | **Ebola Virus Pathway on Host WP4217** | **0.002377** | **0.5609** | **2.91** | **17.61** | ***HLA-DRB5;HLA-B;HLA-DRA;HLA-A;HLA-F;HLA-G;HLA-DQA2;HLA-DQA1;HLA-DRB1;HLA-DQB1*** |
| **3** | **Metapathway biotransformation Phase I and II WP702** | **0.01004** | **1.000** | **2.26** | **10.40** | ***SULT1A1;GSTM4;GLYATL2;GSTM1;CYP2D6;UGT2B15;UGT2B17;GSTT2;UGT2B28;CYP2F1;GSTT2B*** |
| **4** | **Tamoxifen metabolism WP691** | **0.01743** | **1.000** | **5.37** | **21.75** | ***SULT1A1;CYP2D6;UGT2B15*** |
| **5** | **Glucuronidation WP698** | **0.03091** | **1.000** | **4.34** | **15.08** | ***UGT2B15;UGT2B28;UGT2B17*** |
| **6** | **Estrogen metabolism WP697** | **0.08164** | **1.000** | **4.18** | **10.47** | ***SULT1A1;GSTM1*** |
| **7** | **Proteasome Degradation WP183** | **0.08280** | **1.000** | **2.43** | **6.04** | ***HLA-B;HLA-A;HLA-F;HLA-G*** |
| **8** | **IL-5 Signaling Pathway WP127** | **0.08976** | **1.000** | **2.82** | **6.80** | ***GSK3A;SHC1;MAPT*** |
| **9** | **Vitamin D Receptor Pathway WP2877** | **0.1135** | **1.000** | **1.65** | **3.60** | ***ORM1;CYP2D6;KRT34;DEFB4A;HLA-DQA2;HLA-DQA1;HLA-DRB1;LCE1D*** |
| **10** | **Glutathione metabolism WP100** | **0.1239** | **1.000** | **3.27** | **6.83** | ***GSTM1;GSTT2*** |
| **Biological Process** | | | | | | |
| **1** | **humoral immune response mediated by circulating immunoglobulin (GO:0002455)** | **6.242e-35** | **3.185e-31** | **12.63** | **994.85** | ***IGHV4-31;IGHV3-20;IGKV2D-30;IGHV3-64;IGHV3-21;IGHV3-43;IGHV3-23;IGHV4-39;IGHV1-24;IGHG4;IGLV3-25;IGHG2;IGLV3-21;IGLC3;TRDC;IGLC2;IGKV1-33;IGHA2;IGHV3-9;IGHV5-51;IGHV4-61;IGHV3-30;IGHV3-33;IGHV3-11;IGHV3-35;IGHV3-13;IGKV3D-20;IGHV3-15;IGHV4-28;IGHV3-38;IGHV3-16;IGKV1D-16;IGHV2-26;IGKV1D-33;IGHV1-18;IGKV1D-12;IGKV2D-28;IGLV2-23;ZP3;IGLV3-19;IGHV1OR21-1;HLA-DQB1*** |
| **2** | **complement activation, classical pathway (GO:0006958)** | **1.138e-32** | **2.903e-29** | **12.23** | **899.25** | ***IGHV4-31;IGHV3-20;IGKV2D-30;IGHV3-64;IGHV3-21;IGHV3-43;IGHV3-23;IGHV4-39;IGHV1-24;IGHG4;IGLV3-25;IGHG2;IGLV3-21;IGLC3;TRDC;IGLC2;IGKV1-33;IGHA2;IGHV3-9;IGHV5-51;IGHV4-61;IGHV3-30;IGHV3-33;IGHV3-11;IGHV3-35;IGHV3-13;IGKV3D-20;IGHV3-15;IGHV4-28;IGHV3-38;IGHV3-16;IGKV1D-16;IGHV2-26;IGKV1D-33;IGHV1-18;IGKV1D-12;IGKV2D-28;IGLV2-23;IGLV3-19;IGHV1OR21-1*** |
| **3** | **positive regulation of lymphocyte activation (GO:0051251)** | **2.887e-24** | **4.911e-21** | **11.21** | **607.38** | ***IGHV4-31;IGHV3-20;IGHV3-64;IGHV3-21;IGHV3-43;IGHV3-23;IGHV4-39;IGHV1-24;SIRPB1;HSPD1;IGHG4;IGHG2;IGLC3;TRDC;IGLC2;IGHA2;CCR2;IGHV5-51;IGHV4-61;IGHV3-30;IGHV3-33;IGHV3-11;IGHV3-35;IGHV3-13;IGHV3-15;IGHV4-28;IGHV3-38;IGHV3-16;IGHV2-26;IGHV1-18;IGHV1OR21-1*** |
| **4** | **regulation of B cell activation (GO:0050864)** | **6.922e-22** | **8.830e-19** | **11.08** | **539.86** | ***IGHV4-31;IGHV3-20;IGHV3-64;IGHV3-21;IGHV3-43;IGHV3-23;IGHV4-39;IGHV1-24;IGHG4;IGHG2;IGLC3;TRDC;IGLC2;IGHA2;IGHV5-51;IGHV4-61;IGHV3-30;IGHV3-33;IGHV3-11;IGHV3-35;IGHV3-13;IGHV3-15;IGHV4-28;IGHV2-26;IGHV3-38;IGHV3-16;IGHV1-18;IGHV1OR21-1*** |
| **5** | **plasma membrane invagination (GO:0099024)** | **8.007e-21** | **8.172e-18** | **10.22** | **472.91** | ***IGHV4-31;IGHV3-20;IGHV3-64;IGHV3-21;IGHV3-43;IGHV3-23;IGHV4-39;IGHV1-24;IGHG4;IGHG2;IGLC3;TRDC;IGLC2;IGHA2;IGHV5-51;IGHV4-61;IGHV3-30;IGHV3-33;IGHV3-11;IGHV3-35;IGHV3-13;IGHV3-15;IGHV4-28;IGHV3-38;IGHV3-16;IGHV2-26;IGHV1-18;IGHV1OR21-1*** |
| **6** | **B cell receptor signaling pathway (GO:0050853)** | **1.069e-20** | **9.091e-18** | **10.12** | **465.44** | ***IGHV4-31;IGHV3-20;IGHV3-64;IGHV3-21;IGHV3-43;IGHV3-23;IGHV4-39;IGHV1-24;IGHG4;IGHG2;IGLC3;TRDC;IGLC2;IGHA2;IGHV5-51;IGHV4-61;IGHV3-30;IGHV3-33;IGHV3-11;IGHV3-35;IGHV3-13;IGHV3-15;IGHV4-28;IGHV3-38;IGHV3-16;IGHV2-26;IGHV1-18;IGHV1OR21-1*** |
| **7** | **phagocytosis, engulfment (GO:0006911)** | **1.069e-20** | **7.792e-18** | **10.12** | **465.44** | ***IGHV4-31;IGHV3-20;IGHV3-64;IGHV3-21;IGHV3-43;IGHV3-23;IGHV4-39;IGHV1-24;IGHG4;IGHG2;IGLC3;TRDC;IGLC2;IGHA2;IGHV5-51;IGHV4-61;IGHV3-30;IGHV3-33;IGHV3-11;IGHV3-35;IGHV3-13;IGHV3-15;IGHV4-28;IGHV3-38;IGHV3-16;IGHV2-26;IGHV1-18;IGHV1OR21-1*** |
| **8** | **positive regulation of B cell activation (GO:0050871)** | **5.571e-19** | **3.554e-16** | **8.85** | **371.80** | ***IGHV4-31;IGHV3-20;IGHV3-64;IGHV3-21;IGHV3-43;IGHV3-23;IGHV4-39;IGHV1-24;IGHG4;IGHG2;IGLC3;TRDC;IGLC2;IGHA2;IGHV5-51;IGHV4-61;IGHV3-30;IGHV3-33;IGHV3-11;IGHV3-35;IGHV3-13;IGHV3-15;IGHV4-28;IGHV3-38;IGHV3-16;IGHV2-26;IGHV1-18;IGHV1OR21-1*** |
| **9** | **antigen receptor-mediated signaling pathway (GO:0050851)** | **7.108e-18** | **4.030e-15** | **5.56** | **219.48** | ***IGHV4-31;IGHV3-20;IGHV3-64;IGHV3-21;IGHV3-43;IGHV3-23;IGHV1-24;IGHV4-39;TRAV29DV5;IGHG4;IGHG2;IGLC3;TRDC;IGLC2;IGHA2;HLA-DQA2;HLA-DQA1;IGHV5-51;BTNL8;IGHV4-61;HLA-DRB5;IGHV3-30;PDPK1;IGHV3-33;IGHV3-11;IGHV3-35;IGHV3-13;IGHV3-15;BTNL3;IGHV4-28;IGHV3-38;IGHV3-16;IGHV2-26;IGHV1-18;HLA-DRA;HLA-DRB1;IGHV1OR21-1;HLA-DQB1*** |
| **10** | **phagocytosis (GO:0006909)** | **1.997e-17** | **1.019e-14** | **7.80** | **299.82** | ***IGHV4-31;IGHV3-20;IGHV3-64;IGHV3-21;IGHV3-43;IGHV3-23;IGHV4-39;IGHV1-24;IGHG4;IGHG2;IGLC3;TRDC;IGLC2;IGHA2;IGHV5-51;IGHV4-61;IGHV3-30;IGHV3-33;IGHV3-11;IGHV3-35;IGHV3-13;IGHV3-15;IGHV4-28;IGHV3-38;IGHV3-16;IGHV2-26;IGHV1-18;IGHV1OR21-1*** |
