# Supplementary material for: Germline copy number variations in BRCA1/2 negative families: Role in the molecular etiology of hereditary breast cancer in Tunisia
Source: PLoS One. 2021 Jan 27;16(1):e0245362. doi: 10.1371/journal.pone.0245362 (PMC7840007; doi:10.1371/journal.pone.0245362)
Supplement: S3 Table — (DOCX) [file pone.0245362.s003.docx]

***S3 Table. Known cancer predisposition genes frequently investigated in Hereditary Breast and Ovarian Cancer families***

| **Gene** | **Locus** | **Reference** |
| --- | --- | --- |
| ***PALB2*** | **16p12.2** | Couch, 2017 ; Buys,2017 ; Susswein,2016 ; Ramus, 2015 ; Slavin,2017 ; Thompson,2016 ; Norquist,2016 ; Couch,2015 ; Tung , 2014 ; Minion , 2015 ; LaDuca,2014 ; Lincoin , 2015 ; Castera , 2014 ; Li,2016; shroeder,2015 ; Shirts,2015 ; Prtizlaff,2017 ; Eliade,2017; Kraus,2017 ; Tung,2016; Pennington,2014 ; Walsh,2011; Lhota,2016; Frey,2015 ; Crawford,2017 ; Churpek,2015; Maxwell,2015 ; Eccles,2016; tedaldi,2017; Kurian,2014 ; Moran, 2017; Hirotsu,2015 ; Cybulski,2015 ; Lin,2016 ; Frey,2017 ; Mannan,2016 ; Aloraifi , 2016 ; Ng,2016 ; Rajkumar,2016 ; Cock-Rada,2017 ; Pinto , 2016 ; Stafford,2017 ; Churpek,2016 ; Spugnesi,2016 ; Feliubadalo,2017 ; Dominguez-Valentin,2017 |
| ***TP53*** | **17p13.1** | Couch, 2017 ; Buys,2017 ; Susswein,2016 ; Slavin,2017 ; Thompson,2016 ; Norquist,2016 ; Couch,2015 ; Tung , 2014 ; Kwong,2016 ; Minion , 2015 ; LaDuca,2014 ; Lincoin , 2015 ; Castera , 2014 ; Li,2016; shroeder,2015 ; Shirts,2015 ; Prtizlaff,2017 ; Eliade,2017; Kraus,2017 ; Tung,2016; Pennington,2014 ; Walsh,2011; Lhota,2016 ; Crawford,2017 ; Churpek,2015; Maxwell,2015 ; Eccles,2016; tedaldi,2017; Kurian,2014 ; Moran, 2017; Hirotsu,2015 ; Cybulski,2015 ; Lin,2016 ; Doherty,2015 ; Frey,2017 ; Mannan,2016 ; Aloraifi , 2016 ; Ng,2016 ; Rajkumar,2016 ; Cock-Rada,2017 ; Pinto , 2016 ; Stafford,2017 ; Churpek,2016 ; Spugnesi,2016 ; Feliubadalo,2017 ; Dominguez-Valentin,2017 |
| ***ATM*** | **11q22.3** | Couch, 2017 ; Buys,2017 ; Susswein,2016 ; Slavin,2017 ; Thompson,2016 ; Norquist,2016 ; Couch,2015 ; Tung , 2014 ; Minion , 2015 ; LaDuca,2014 ; Lincoin , 2015 ; Castera , 2014 ; Li,2016; shroeder,2015 ; Shirts,2015 ; Prtizlaff,2017 ; Eliade,2017; Kraus,2017 ; Tung,2016; Pennington,2014 ; Walsh,2011; Lhota,2016; Frey,2015 ; Crawford,2017 ; Churpek,2015; Maxwell,2015 ; Eccles,2016; tedaldi,2017; Kurian,2014 ; Moran, 2017; Hirotsu,2015 ; Cybulski,2015 ; Lin,2016 ; Frey,2017 ; Mannan,2016 ; Aloraifi , 2016 ; Ng,2016 ; Rajkumar,2016 ; Cock-Rada,2017 ; Pinto , 2016 ; Stafford,2017 ; Churpek,2016 ; Feliubadalo,2017 ; Dominguez-Valentin,2017 |
| ***BRCA1*** | **17q21.31** | Couch, 2017 ; Buys,2017 ; Susswein,2016 ; Thompson,2016 ; Norquist,2016 ; Couch,2015 ; Tung , 2014 ; Kwong,2016 ; Minion , 2015 ; Lincoin , 2015 ; Castera , 2014 ; Li,2016; shroeder,2015 ; Shirts,2015 ; Prtizlaff,2017 ; Eliade,2017; Kraus,2017 ; Tung,2016; Pennington,2014 ; Walsh,2011; Lhota,2016; Frey,2015 ; Crawford,2017 ; Churpek,2015; Maxwell,2015 ; Eccles,2016; tedaldi,2017; Kurian,2014 ; Moran, 2017 ; Cybulski,2015 ; Lin,2016 ; Doherty,2015 ; Frey,2017 ; Mannan,2016 ; Aloraifi , 2016 ; Ng,2016 ; Rajkumar,2016 ; Cock-Rada,2017 ; Pinto , 2016 ; Stafford,2017 ; Churpek,2016 ; Spugnesi,2016 ; Feliubadalo,2017 ; Dominguez-Valentin,2017 |
| ***BRCA2*** | **13q13.1** | Couch, 2017 ; Buys,2017 ; Susswein,2016 ; Thompson,2016 ; Norquist,2016 ; Couch,2015 ; Tung , 2014 ; Kwong,2016 ; Minion , 2015 ; Lincoin , 2015 ; Castera , 2014 ; Li,2016; shroeder,2015 ; Shirts,2015 ; Prtizlaff,2017 ; Eliade,2017; Kraus,2017 ; Tung,2016; Pennington,2014 ; Walsh,2011; Lhota,2016; Frey,2015 ; Crawford,2017 ; Churpek,2015; Maxwell,2015 ; Eccles,2016; tedaldi,2017; Kurian,2014 ; Moran, 2017 ; Cybulski,2015 ; Lin,2016 ; Doherty,2015 ; Frey,2017 ; Mannan,2016 ; Aloraifi , 2016 ; Ng,2016 ; Rajkumar,2016 ; Cock-Rada,2017 ; Pinto , 2016 ; Stafford,2017 ; Churpek,2016 ; Spugnesi,2016 ; Feliubadalo,2017 ; Dominguez-Valentin,2017 |
| ***CHEK2*** | **22q12.1** | Couch, 2017 ; Buys,2017 ; Susswein,2016 ; Slavin,2017 ; Thompson,2016 ; Norquist,2016 ; Couch,2015 ; Tung , 2014 ; Minion , 2015 ; LaDuca,2014 ; Lincoin , 2015 ; Castera , 2014 ; Li,2016; shroeder,2015 ; Shirts,2015 ; Prtizlaff,2017 ; Eliade,2017; Kraus,2017 ; Tung,2016; Pennington,2014 ; Walsh,2011; Lhota,2016; Frey,2015 ; Crawford,2017 ; Churpek,2015; Maxwell,2015 ; Eccles,2016; tedaldi,2017 ; Moran, 2017; Hirotsu,2015 ; Cybulski,2015 ; Lin,2016 ; Frey,2017 ; Mannan,2016 ; Aloraifi , 2016 ; Ng,2016 ; Rajkumar,2016 ; Cock-Rada,2017 ; Pinto , 2016 ; Stafford,2017 ; Churpek,2016 ; Spugnesi,2016 ; Feliubadalo,2017 ; Dominguez-Valentin,2017 |
| ***PTEN*** | **10q23.31** | Couch, 2017 ; Buys,2017 ; Susswein,2016 ; Slavin,2017 ; Thompson,2016 ; Norquist,2016 ; Couch,2015 ; Tung , 2014 ; Kwong,2016 ; Minion , 2015 ; LaDuca,2014 ; Lincoin , 2015 ; Castera , 2014 ; Li,2016 ; Shirts,2015 ; Prtizlaff,2017 ; Eliade,2017 ; Tung,2016; Pennington,2014 ; Walsh,2011; Lhota,2016; Frey,2015 ; Crawford,2017 ; Churpek,2015; Maxwell,2015 ; Eccles,2016; tedaldi,2017; Kurian,2014 ; Moran, 2017; Hirotsu,2015 ; Cybulski,2015 ; Lin,2016 ; Doherty,2015 ; Mannan,2016 ; Aloraifi , 2016 ; Ng,2016 ; Rajkumar,2016 ; Cock-Rada,2017 ; Pinto , 2016 ; Stafford,2017 ; Churpek,2016 ; Spugnesi,2016 ; Feliubadalo,2017 ; Dominguez-Valentin,2017 |
| ***NBN*** | **8q21.3** | Couch, 2017 ; Buys,2017 ; Susswein,2016 ; Ramus, 2015 ; Slavin,2017 ; Thompson,2016 ; Norquist,2016 ; Couch,2015 ; Tung , 2014 ; Minion , 2015 ; LaDuca,2014 ; Lincoin , 2015 ; Castera , 2014 ; Li,2016; shroeder,2015 ; Shirts,2015 ; Prtizlaff,2017 ; Kraus,2017 ; Tung,2016; Pennington,2014 ; Walsh,2011; Lhota,2016 ; Crawford,2017 ; Maxwell,2015 ; Eccles,2016; tedaldi,2017; Kurian,2014 ; Moran, 2017; Hirotsu,2015 ; Cybulski,2015 ; Lin,2016 ; Doherty,2015 ; Mannan,2016 ; Aloraifi , 2016 ; Ng,2016 ; Rajkumar,2016 ; Cock-Rada,2017 ; Pinto , 2016 ; Stafford,2017 ; Churpek,2016 ; Spugnesi,2016 ; Feliubadalo,2017 ; Dominguez-Valentin,2017 |
| ***BRIP1*** | **17q23.2** | Couch, 2017 ; Buys,2017 ; Susswein,2016 ; Ramus, 2015 ; Slavin,2017 ; Thompson,2016 ; Norquist,2016 ; Couch,2015 ; Tung , 2014 ; Minion , 2015 ; LaDuca,2014 ; Lincoin , 2015 ; Castera , 2014 ; Li,2016 ; Shirts,2015 ; Prtizlaff,2017 ; Eliade,2017 ; Tung,2016; Pennington,2014 ; Walsh,2011; Lhota,2016; Frey,2015 ; Crawford,2017 ; Maxwell,2015 ; Eccles,2016; tedaldi,2017; Kurian,2014 ; Hirotsu,2015 ; Cybulski,2015 ; Lin,2016 ; Frey,2017 ; Mannan,2016 ; Aloraifi , 2016 ; Ng,2016 ; Rajkumar,2016 ; Cock-Rada,2017 ; Pinto , 2016 ; Stafford,2017 ; Churpek,2016 ; Spugnesi,2016 ; Feliubadalo,2017 ; Dominguez-Valentin,2017 |
| ***CDH1*** | **16q22.1** | Couch, 2017 ; Buys,2017 ; Slavin,2017 ; Thompson,2016 ; Couch,2015 ; Tung , 2014 ; Minion , 2015 ; LaDuca,2014 ; Lincoin , 2015 ; Castera , 2014 ; Li,2016; shroeder,2015 ; Shirts,2015 ; Prtizlaff,2017 ; Eliade,2017; Kraus,2017 ; Tung,2016; Pennington,2014 ; Walsh,2011; Lhota,2016 ; Crawford,2017 ; Maxwell,2015 ; Eccles,2016; tedaldi,2017; Kurian,2014 ; Hirotsu,2015 ; Lin,2016 ; Doherty,2015 ; Frey,2017 ; Mannan,2016 ; Aloraifi , 2016 ; Ng,2016 ; Rajkumar,2016 ; Cock-Rada,2017 ; Pinto , 2016 ; Stafford,2017 ; Churpek,2016 ; Spugnesi,2016 ; Feliubadalo,2017 ; Dominguez-Valentin,2017 |
| ***BARD1*** | **2q35** | Couch, 2017 ; Buys,2017 ; Susswein,2016 ; Ramus, 2015 ; Slavin,2017 ; Thompson,2016 ; Norquist,2016 ; Couch,2015 ; Tung , 2014 ; Minion , 2015 ; LaDuca,2014 , 2015 ; Castera , 2014 ; Li,2016 ; Shirts,2015 ; Prtizlaff,2017 ; Eliade,2017 ; Tung,2016; Pennington,2014 ; Walsh,2011; Lhota,2016; Frey,2015 ; Crawford,2017 ; Churpek,2015; Maxwell,2015 ; Eccles,2016 ; Moran, 2017; Hirotsu,2015 ; Cybulski,2015 ; Lin,2016 ; Frey,2017 ; Aloraifi , 2016 ; Ng,2016 ; Rajkumar,2016 ; Cock-Rada,2017 ; Stafford,2017 ; Churpek,2016 ; Spugnesi,2016 ; Feliubadalo,2017 ; Dominguez-Valentin,2017 |
| ***RAD51C*** | **17q22** | Couch, 2017 ; Buys,2017 ; Susswein,2016 ; Slavin,2017 ; Norquist,2016 ; Couch,2015 ; Tung , 2014 ; Minion , 2015 ; LaDuca,2014 ; Lincoin , 2015 ; Castera , 2014 ; Li,2016; shroeder,2015 ; Shirts,2015 ; Prtizlaff,2017 ; Eliade,2017; Kraus,2017 ; Tung,2016; Pennington,2014 ; Walsh,2011; Lhota,2016 ; Crawford,2017 ; Churpek,2015; Maxwell,2015 ; tedaldi,2017; Kurian,2014 ; Hirotsu,2015 ; Lin,2016 ; Mannan,2016 ; Aloraifi , 2016 ; Ng,2016 ; Rajkumar,2016 ; Cock-Rada,2017 ; Pinto , 2016 ; Stafford,2017 ; Churpek,2016 ; Spugnesi,2016 ; Feliubadalo,2017 ; Dominguez-Valentin,2017 |
| ***STK11*** | **19p13.3** | Buys,2017 ; Slavin,2017 ; Thompson,2016 ; Couch,2015 ; Tung , 2014 ; Minion , 2015 ; LaDuca,2014 ; Lincoin , 2015 ; Castera , 2014 ; Li,2016 ; Shirts,2015 ; Eliade,2017 ; Tung,2016; Pennington,2014 ; Walsh,2011; Lhota,2016 Crawford,2017 ; Maxwell,2015 ; Eccles,2016; tedaldi,2017; Kurian,2014 ; Hirotsu,2015 ; Lin,2016 ; Doherty,2015 ; Mannan,2016 ; Aloraifi , 2016 ; Ng,2016 ; Rajkumar,2016 ; Cock-Rada,2017 ; Pinto , 2016 ; Stafford,2017 ; Churpek,2016 ; Spugnesi,2016 ; Feliubadalo,2017 ; Dominguez-Valentin,2017 |
| ***MLH1*** | **19p13.3** | Couch, 2017 ; Buys,2017 ; Susswein,2016 ; Slavin,2017 ; Norquist,2016 ; Tung , 2014 ; Minion , 2015 ; Lincoin , 2015 ; Castera , 2014 ; Shirts,2015 ; Eliade,2017; Kraus,2017 ; Tung,2016; Pennington,2014 ; Walsh,2011; Lhota,2016; Frey,2015 ; Crawford,2017 ; Maxwell,2015 ; tedaldi,2017; Kurian,2014 ; Hirotsu,2015 ; Lin,2016 ; Frey,2017 ; Aloraifi , 2016 ; Rajkumar,2016 ; Cock-Rada,2017 ; Pinto , 2016 ; Stafford,2017 ; Churpek,2016 ; Spugnesi,2016 ; Feliubadalo,2017 ; Dominguez-Valentin,2017 |
| ***MSH6*** | **2p16.3** | Couch, 2017 ; Buys,2017 ; Susswein,2016 ; Slavin,2017 ; Norquist,2016 ; Tung , 2014 ; Minion , 2015 ; Lincoin , 2015 ; Castera , 2014 ; Shirts,2015 ; Eliade,2017; Kraus,2017 ; Tung,2016; Pennington,2014 ; Walsh,2011; Lhota,2016; Frey,2015 ; Crawford,2017 ; Maxwell,2015 ; tedaldi,2017; Kurian,2014 ; Hirotsu,2015 ; Lin,2016 ; Frey,2017 ; Aloraifi , 2016 ; Rajkumar,2016 ; Cock-Rada,2017 ; Pinto , 2016 ; Stafford,2017 ; Churpek,2016 ; Spugnesi,2016 ; Feliubadalo,2017 ; Dominguez-Valentin,2017 |
| ***MSH2*** | **2p21** | Couch, 2017 ; Buys,2017 ; Susswein,2016 ; Slavin,2017 ; Norquist,2016 ; Tung , 2014 ; Minion , 2015 ; Lincoin , 2015 ; Castera , 2014 ; Shirts,2015 ; Eliade,2017; Kraus,2017 ; Tung,2016; Pennington,2014 ; Walsh,2011; Lhota,2016 ; Crawford,2017 ; Maxwell,2015 ; tedaldi,2017; Kurian,2014 ; Hirotsu,2015 ; Lin,2016 ; Frey,2017 ; Aloraifi , 2016 ; Rajkumar,2016 ; Cock-Rada,2017 ; Pinto , 2016 ; Stafford,2017 ; Churpek,2016 ; Spugnesi,2016 ; Feliubadalo,2017 ; Dominguez-Valentin,2017 |
| ***PMS2*** | **7p22.1** | Couch, 2017 ; Buys,2017 ; Susswein,2016 ; Slavin,2017 ; Norquist,2016 ; Tung , 2014 ; Minion , 2015 ; Lincoin , 2015 ; Castera , 2014 ; Shirts,2015 ; Eliade,2017; Kraus,2017 ; Tung,2016; Pennington,2014 ; Walsh,2011; Lhota,2016; Frey,2015 ; Crawford,2017 ; Maxwell,2015 ; tedaldi,2017; Kurian,2014 ; Hirotsu,2015 ; Lin,2016 ; Aloraifi , 2016 ; Rajkumar,2016 ; Cock-Rada,2017 ; Pinto , 2016 ; Stafford,2017 ; Churpek,2016 ; Spugnesi,2016 ; Feliubadalo,2017 ; Dominguez-Valentin,2017 |
| ***RAD51D*** | **17q12** | Couch, 2017 ; Buys,2017 ; Susswein,2016 ; Slavin,2017 ; Norquist,2016 ; Couch,2015 ; Tung , 2014 ; Castera , 2014 ; Li,2016; shroeder,2015 ; Shirts,2015 ; Prtizlaff,2017 ; Kraus,2017 ; Tung,2016 ; Lhota,2016; Frey,2015 ; Crawford,2017 ; Churpek,2015; tedaldi,2017 ; Hirotsu,2015 ; Lin,2016 ; Frey,2017 ; Mannan,2016 ; Aloraifi , 2016 ; Cock-Rada,2017 ; Pinto , 2016 ; Stafford,2017 ; Churpek,2016 ; Feliubadalo,2017 ; Dominguez-Valentin,2017 |
| ***MUTYH*** | **1p34.1** | Buys,2017 ; Susswein,2016 ; Slavin,2017 ; Tung , 2014 ; Minion , 2015 ; LaDuca,2014 ; Lincoin , 2015 ; Shirts,2015 ; Eliade,2017 ; Tung,2016; Pennington,2014 ; Walsh,2011; Lhota,2016; Frey,2015 ; Maxwell,2015 ; tedaldi,2017; Kurian,2014 ; Hirotsu,2015 ; Lin,2016 ; Frey,2017 ; Aloraifi , 2016 ; Rajkumar,2016 ; Cock-Rada,2017 ; Stafford,2017 ; Churpek,2016 ; Spugnesi,2016 ; Feliubadalo,2017 ; Dominguez-Valentin,2017 |
| ***MRE11A*** | **11q21** | Couch, 2017 ; Slavin,2017 ; Thompson,2016 ; Norquist,2016 ; Couch,2015 ; Minion , 2015 ; LaDuca,2014 ; Castera , 2014 ; Li,2016 ; Shirts,2015 ; Prtizlaff,2017 ; Eliade,2017 ; Pennington,2014 ; Walsh,2011; Lhota,2016 ; Maxwell,2015 ; Eccles,2016; Hirotsu,2015 ; Lin,2016 ; Frey,2017 ; Aloraifi , 2016 ; Ng,2016 ; Rajkumar,2016 ; Churpek,2016 ; Spugnesi,2016 ; Feliubadalo,2017 ; Dominguez-Valentin,2017 |
| ***RAD50*** | **5q31.1** | Couch, 2017 ; Slavin,2017 ; Thompson,2016 ; Norquist,2016 ; Couch,2015 ; Minion , 2015 ; LaDuca,2014 ; Castera , 2014 ; Li,2016 ; Shirts,2015 ; Prtizlaff,2017 ; Eliade,2017 ; Pennington,2014 ; Walsh,2011; Lhota,2016 ; Maxwell,2015 ; Eccles,2016; Hirotsu,2015 ; Lin,2016 ; Frey,2017 ; Aloraifi , 2016 ; Ng,2016 ; Rajkumar,2016 ; Stafford,2017 ; Churpek,2016 ; Spugnesi,2016 ; Feliubadalo,2017 |
| ***APC*** | **5q22.2** | Buys,2017 ; Susswein,2016 ; Tung , 2014 ; Lincoin , 2015 ; Shirts,2015 ; Prtizlaff,2017 ; Eliade,2017 ; Tung,2016; Pennington,2014 ; Lhota,2016; Frey,2015 ; tedaldi,2017; Kurian,2014 ; Hirotsu,2015 ; Lin,2016 ; Frey,2017 ; Aloraifi , 2016 ; Cock-Rada,2017 ; Stafford,2017 ; Churpek,2016 ; Feliubadalo,2017 ; Dominguez-Valentin,2017 |
| ***CDKN2A*** | **9p21.3** | Couch, 2017 ; Buys,2017 ; Tung , 2014 ; Lincoin , 2015 ; Li,2016 ; Shirts,2015 ; Tung,2016; Pennington,2014 ; Lhota,2016; Frey,2015 ; Maxwell,2015 ; tedaldi,2017; Kurian,2014 ; Hirotsu,2015 ; Frey,2017 ; Aloraifi , 2016 ; Cock-Rada,2017 ; Stafford,2017 ; Churpek,2016 ; Feliubadalo,2017 ; Dominguez-Valentin,2017 |
| ***EPCAM*** | **2p21** | Buys,2017 ; Tung , 2014 ; Minion , 2015 ; Lincoin , 2015 ; Shirts,2015 ; Eliade,2017; Tung,2016; Pennington,2014 ; Crawford,2017 ; tedaldi,2017; Kurian,2014 ; Hirotsu,2015 ; Lin,2016 ; Frey,2017 ; Aloraifi , 2016 ; Cock-Rada,2017 ; Stafford,2017 ; Churpek,2016 ; Feliubadalo,2017 ; Dominguez-Valentin,2017 |
| ***SMAD4*** | **18q21.2** | Buys,2017 ; Tung , 2014 ; Lincoin , 2015 ; Shirts,2015 ; Tung,2016; Pennington,2014 ; tedaldi,2017; Kurian,2014 ; Hirotsu,2015 ; Lin,2016 ; Frey,2017 ; Aloraifi , 2016 ; Cock-Rada,2017 ; Stafford,2017 ; Churpek,2016 ; Feliubadalo,2017 ; Dominguez-Valentin,2017 |
| ***BMPR1A*** | **10q23.2** | Buys,2017 ; Tung , 2014 ; Lincoin , 2015 ; Shirts,2015 ; Tung,2016; Pennington,2014 ; tedaldi,2017; Kurian,2014 ; Hirotsu,2015 ; Lin,2016 ; Frey,2017 ; Cock-Rada,2017 ; Stafford,2017 ; Churpek,2016 ; Feliubadalo,2017 ; Dominguez-Valentin,2017 |
| ***XRCC2*** | **7q36.1** | Susswein,2016 ; Slavin,2017 ; Thompson,2016 ; Couch,2015 ; Castera , 2014 ; Li,2016 ; Shirts,2015 ; Lhota,2016 ; Moran, 2017 ; Cybulski,2015 ; Lin,2016 ; Frey,2017 ; Stafford,2017 ; Churpek,2016 ; Feliubadalo,2017 ; Dominguez-Valentin,2017 |
| ***CDK4*** | **12q14.1** | Buys,2017 ; Tung , 2014 ; Lincoin , 2015 ; Li,2016 ; Shirts,2015 ; Tung,2016; Pennington,2014 ; Lhota,2016 ; tedaldi,2017; Kurian,2014 ; Hirotsu,2015 ; Cock-Rada,2017 ; Stafford,2017 ; Churpek,2016 ; Feliubadalo,2017 |
| ***BLM*** | **15q26.1** | Slavin,2017 ; Thompson,2016 ; Lhota,2016; Eccles,2016; tedaldi,2017; Kurian,2014 ; Moran, 2017 ; Cybulski,2015 ; Frey,2017 ; Stafford,2017 ; Feliubadalo,2017 |
| ***FANCC*** | **9q22.32** | Susswein,2016 ; Slavin,2017 ; Lhota,2016; Frey,2015 ; tedaldi,2017; Kurian,2014 ; Lin,2016 ; Aloraifi , 2016 ; Stafford,2017 ; Feliubadalo,2017 |
| ***NF1*** | **17q11.2** | Couch, 2017 ; Thompson,2016 ; Prtizlaff,2017 ; Lhota,2016 ; Eccles,2016; tedaldi,2017 ; Frey,2017 ; Aloraifi , 2016 ; Ng,2016 ; Feliubadalo,2017 |
| ***BAP1*** | **3p21.1** | Slavin,2017 ; Castera , 2014 ; Shirts,2015 ; Pennington,2014 ; Lhota,2016; tedaldi,2017; Churpek,2016 ; Feliubadalo,2017 ; Dominguez-Valentin,2017 |
| ***ATR*** | **3q23** | Thompson,2016 ; Shirts,2015 ; Lhota,2016; Eccles,2016; Aloraifi , 2016 ; Rajkumar,2016 ; Stafford,2017 ; Churpek,2016 |
| ***VHL*** | **3p25.3** | Susswein,2016; Lincoin , 2015 ; Shirts,2015 ; Pennington,2014 ; tedaldi,2017; Kurian,2014 ; Churpek,2016 ; Feliubadalo,2017 |
| ***FAM175A*** | **4q21.23** | Norquist,2016; Shirts,2015; Lhota,2016; Frey,2015 ; Maxwell,2015 ; Churpek,2016 ; Dominguez-Valentin,2017 |
| ***FANCM*** | **14q21.2** | Slavin,2017 ; Lhota,2016; tedaldi,2017; Lin,2016 ; Aloraifi , 2016 ; Stafford,2017 ; Feliubadalo,2017 |
| ***SLX4*** | **16p13.3** | Norquist,2016 ; Lhota,2016; tedaldi,2017; Kurian,2014 ; Lin,2016 ; Stafford,2017 ; Feliubadalo,2017 |
